# Targeted Drug Delivery Systems Mediated by a Novel Peptide in Breast Cancer Therapy and Imaging

**DOI:** 10.1371/journal.pone.0066128

**Published:** 2013-06-11

**Authors:** Ruei-Min Lu, Min-Shan Chen, De-Kuan Chang, Chien-Yu Chiu, Wei-Chuan Lin, Shin-Long Yan, Yi-Ping Wang, Yuan-Sung Kuo, Chen-Yun Yeh, Albert Lo, Han-Chung Wu

**Affiliations:** 1 Institute of Cellular and Organismic Biology, Academia Sinica, Taipei, Taiwan; 2 Graduate Institute of Oral Biology, College of Medicine, National Taiwan University, Taipei, Taiwan; 3 Department of Surgery, National Taiwan University Hospital, National Taiwan University, Taipei, Taiwan; Cedars-Sinai Medical Center, United States of America

## Abstract

Targeted delivery of drugs to tumors represents a significant advance in cancer diagnosis and therapy. Therefore, development of novel tumor-specific ligands or pharmaceutical nanocarriers is highly desirable. In this study, we utilized phage display to identify a new targeting peptide, SP90, which specifically binds to breast cancer cells, and recognizes tumor tissues from breast cancer patients. We used confocal and electron microscopy to reveal that conjugation of SP90 with liposomes enables efficient delivery of drugs into cancer cells through endocytosis. Furthermore, *in vivo* fluorescent imaging demonstrated that SP90-conjugated quantum dots possess tumor-targeting properties. In tumor xenograft and orthotopic models, SP90-conjugated liposomal doxorubicin was found to improve the therapeutic index of the chemotherapeutic drug by selectively increasing its accumulation in tumors. We conclude that the targeting peptide SP90 has significant potential in improving the clinical benefits of chemotherapy in the treatment and the diagnosis of breast cancer.

## Introduction

Breast cancer is the most common malignancy among women, and the second leading cause of cancer deaths in the United States [1]. The 5-year survival rate for women diagnosed with metastatic breast cancer is only about 27.4% [2]. Although adjuvant chemotherapy, as well as radiation, hormonal, and targeted therapy, have all been used to treat different stages of breast cancer, chemotherapy continues to be the major therapeutic option for patients with metastatic breast cancer [3]. While many cytotoxic drugs (including doxorubicin, vinorelbine, gemcitabine, *nab*-paclitaxel, pemetrexed, platinum salts, etoposide, and irinotecan) have been developed for the treatment of metastatic breast cancer, the response rates for these chemotherapeutic agents are usually poor, and the frequency at which patients develop drug-resistance remains high [4].

One major problem with chemotherapy for the treatment of cancer is the lack of selective toxicity, which results in a narrow therapeutic index, and thereby compromises clinical prognosis. In order to reduce damage to normal tissues, sub-optimal doses of anticancer chemotherapeutics are often administered. Additionally, the high interstitial fluid pressure (IFP) of solid tumors results in poor biodistribution and penetration of drugs [5], [6]. It has been shown that the amount of drug accumulated in normal viscera is ∼10- to 20-fold higher than that in the same weight of tumor site [7], [8], and that many anticancer drugs are not able to penetrate more than 40–50 µm (equivalent to the combined diameter of 3–5 cells) from the vasculature [9], [10]. These defects often lead to incomplete tumor response, multiple drug resistance, and ultimately, therapeutic failure [11].

Nanotechnology holds significant promise for circumventing these challenges, by enabling large amounts of therapeutic drugs to be encapsulated into nanoparticles. This simultaneously increases the half-life and reduces toxic adverse effects of drugs, improving their pharmacokinetic profile and therapeutic efficacy [12]–[14]. Such therapeutic nanoparticles have been broadly employed in cancer drug delivery, and have been found to result in higher drug accumulation in tumors through passive targeting, or the enhanced permeability and retention (EPR) effect, which arises as a consequence of leaky vasculature and inefficient lymphatic drainage in the tumor [15], [16]. There are more than twenty therapeutic nanomaterials that have been approved for clinical use [17]. Of these, lipid-based formulations, such as liposomal doxorubicin (Doxil/Caelyx) and liposomal daunorubicin (DaunoXome), are considered to be a major class of nanocarriers for use in cancer therapy [18]. More recently, albumin has begun to emerge as an ideal protein vehicle for drug delivery. From a physiological perspective, albumin’s long serum half-life (19 days on average) and its ability to carry various protein and hydrophobic molecules (such as vitamins and hormones) throughout the body, make it a suitable candidate for nanoparticle carrier design [19]. One such nanoparticle, albumin-bound paclitaxel (Abraxane), has a diameter comparable to that of liposomal drugs (130 nm), and was approved by the FDA for the treatment of metastatic breast cancer in 2005 [20]. However, not all solid tumors exhibit the EPR effect [15], [16], and nanoparticles are no more efficacious than free drugs in the passive targeting of blood cancers [21].

Therapeutic efficacy and the regulation of drug release can be improved by using selective targeting drug delivery systems. This approach utilizes ligands that specifically interact with receptors expressed on cancer cells, in order to enhance binding and internalization of nanoparticle-based drugs [22]–[25]. Nanoparticles with targeting moieties on their external surface exhibit much higher affinity for cancer cells [26]–[28]. To date, studies on targeted drug delivery for breast cancer have largely centered on pre-clinical studies using anti-HER2 antibody-linked liposomal drugs [29], [30]. However, few reports have studied the use of peptides for targeted drug delivery in breast cancer treatment [31], [32].

Phage display is an efficient tool for rapid selection of ligands for various molecular targets [33]. Using this method, we have previously identified several peptides and antibodies which target tumors and tumor vasculature [8], [34], [35]. In the current study, we use phage display to identify a new peptide that binds to breast cancer cells, as a means of improving drug delivery for therapy and early diagnosis. Furthermore, we outline the proof-of-concept studies in murine animal models to investigate the therapeutic and diagnostic potential of nanoparticles linked to the novel peptide.

## Materials and Methods

### Cell Lines and Cultures

Breast cancer cell lines, BT483, MDA-MB-231, MD1-MB-361, MCF-7 and SK-BR-3, were obtained from the American Type Culture Collection. BT483 cells were grown in DMEM (Invitrogen) containing 20 mM L-glutamine. MDA-MB-231, MD1-MB-361 and SK-BR-3 cells were cultured in DMEM/F12 (Invitrogen), Leibovitz’s L-15 medium (Invitrogen) and McCoy's 5A medium (Sigma-Aldrich), respectively. The human normal nasal mucosal epithelial (NNM) cells were a primary culture derived from a nasal polyp [36] and were grown in DMEM. Normal mammary epithelial cells (HMEpiC) were purchased from ScienCell Research Laboratories and were cultured in Mammary Epithelial Cell Medium (ScienCell Research Laboratories). All cell lines were cultured in a condition containing 10% fetal bovine serum (Invitrogen) and 100 µg/ml penicillin/streptomycin in a humidified incubator with 5% CO_2_ at 37°C.

### Phage Display Biopanning Procedures

Breast cancer cell line BT483 cells were incubated with UV-treated inactive control helper phage (insertless phage). The phage-displayed peptide library (New England BioLabs), which initially contained 5 × 10^10^ plaque-forming units (pfu), was then added. After washing, the bound phages were eluted with a lysis buffer (150 mM NaCl, 50 mM Tris-HCl, 1 mM EDTA, 1% NP-40, 0.5% sodium deoxycholate, 0.1% SDS, pH 7.4) on ice. This eluted phage pool was amplified and titered in an *Escherichia coli* ER2738 culture (New England BioLabs). Recovered phages were used as input for the next round of panning as described previously [36].

### Identification of Phage Clones using Cellular Enzyme-linked Immunosorbent Assay (ELISA)

Ninety-six-well ELISA plates were seeded with either cancer or human normal nasal mucosal epithelial (NNM) control cells. Individual phage particles were added to the cell-coated plates and were incubated with horseradish peroxidase (HRP)-conjugated mouse anti-M13 monoclonal antibody (GE Healthcare), followed by incubating with the peroxidase substrate *o*-phenylenediamine dihydrochloride (Sigma-Aldrich). The reaction was stopped and absorbance was measured at 490 nm using an ELISA reader.

### Sequencing of Recovered Phages

The selected phage clones were further analyzed using DNA sequencing with the primer 5′-CCCTCATAGTTAGCGTAACG-3′ corresponding to the pIII gene sequence. The phage clones with higher BT483-binding activities, PC34, PC65, PC73, PC82 and PC90, displayed QNIYAGVPMISF, EATNSHGSRTMG, TVSWSTTGRIPL, QLEFYTQLAHLI and SMDPFLFQLLQL peptide sequence, respectively. The peptide sequences of PC34, PC65, PC73, PC82 and PC90 have submitted to GenBank and the accession numbers are KC802225, KC802226, KC802227, KC802228 and KC802229, respectively.

### Flow Cytometry Analysis

The breast cancer cell lines or control cells were collected using PBS containing 10 mM EDTA, and then were incubated with 1×10^10^ pfu/mL PC90 phages or insert-less control phages at 4°C for 1 hour. After washing, the phage-bound cells were incubated with anti-M13 mAbs (GE Healthcare) at 4°C for 1 hour and then treated with PE-conjugated goat anti-mouse IgG antibody at 4°C for 30 minutes. Cells were washed and analyzed by flow cytometer (Becton Dickinson).

### Peptide Synthesis

The synthetic targeting peptide SP90 (SMDPFLFQLLQL) and control peptide (MP5-2, TDSILRSYDGGG) [8] were synthesized and purified using reverse-phase high-performance liquid chromatography to 95% purity by Academia Sinica (Taipei, Taiwan).

### *In vivo* Homing Experiments and Tissue Distribution of Phages

SCID mice were injected subcutaneously in the dorsolateral flank with 1 × 10^7^ BT483 cells. The mice bearing size-matched breast cancer xenografts (approximately 300 mm^3^) were intravenously administered with 10^9^ pfu of the targeting phage or control phage. After eight minutes of phage circulation, the mice were sacrificed and perfused with 50 ml PBS to wash out unbound phage. Subsequently, xenograft tumors and mouse organs were dissected and homogenized. The phages bound to each tissue sample were recovered by adding ER2738 bacteria and titered on IPTG/X-Gal agar plates. In the peptide competitive inhibition experiments, the phages were injected along with 100 µg synthetic targeting peptide. The organs and tumor masses were fixed in Bouin’s solution (Sigma-Aldrich). After fixation, the samples were embedded in paraffin blocks. The paraffin sections were deparaffinized, rehydrated, and subjected to immunostaining using the mouse anti- M13 mAb.

### Immunohistochemistry Staining for Human Surgical Specimens

A total of twenty cases of frozen tissue blocks consisted of infiltrating ducal carcinoma of the breast were obtained from tissue bank of National Taiwan University Hospital (NTUH) with approval from the Institutional Review Board in NTUH (IRB9461702021). Each tissue section was cut at 4 µm and fixed in 1% paraformadehyde. For localization of phages binding to the breast cancer tissues, the tissues were incubated with PC90 or control phages. For the peptide competitive inhibition assay, the phages were mixed with 100 ng/ml synthetic SP90 or control peptides. After washing in PBS with 0.1% Tween 20 (PBST0.1), sections were treated with anti-M13 mouse mAb (GE Healthcare) for 1 hour at room temperature. Following washing in PBST0.1, a biotin-free super sensitive polymer-HRP detection system (Biogenex) was used to detect immunoreactivity. The slides were lightly counterstained with hematoxylin, mounted with Aquatex (Merck) and examined by light microscopy. The percentage of positive staining cells was quantified and labeling index (LI) of each section was judged by the medical pathologist of NTUH.

### Synthesis of SP90-PEG-DSPE Conjugates

8.5 mg of NHS-PEG-DSPE [N-hydroxysuccinimido-carboxyl-polyethylene glycol (MW, 3400)-derived distearoylphosphatidyl ethanolamine] (NOF Corporation) dissolved in 0.25 ml of dichloromethane (Sigma-Aldrich) was added to 0.25 ml of DMSO (Sigma-Aldrich) containing 3.1 mg of SP90 peptide. This was then mixed with 0.011 ml of triethylamine (Sigma-Aldrich) to catalyze the reaction. The stoichiometric molar ratio of SP90 and NHS-PEG-DSPE was 1.1∶1. The reaction was carried out for 72 hours at room temperature. The SP90-PEG-DSPE conjugates were purified by dialysis with a 2 kDa cut-off membrane (Spectrum), and were then dried through lyophilization.

To determine the percentage of conjugation between PEG and SP90, equal concentrations of SP90, NHS-PEG-DSPE and SP90-PEG-DSPE conjugates prior to purification were subjected to electrophoresis on a Tricine-SDS polyacrylamide gel (20% acrylamide; 6% crosslinker, relative to the total concentration), as modified from a previous paper [37]. Precision Plus Protein Dual Xtra Standards (Bio-Rad) were used as protein markers. Coomassie blue and barium iodide staining were used to detect peptide and PEG molecules, respectively. After Coomassie blue staining, gels were rinsed with ddH_2_O, and immersed in 5% (w/v) barium chloride solution (Sigma-Aldrich) for 10 min. After a second rinse with ddH_2_O, the gel was placed in 0.05 M iodine solution (Sigma-Aldrich) for color development. The conjugate was also analyzed by MALDI-TOF-MS (BRUKER microflex) operated in the linear mode. The matrix used was α-cyano-4-hydroxycinnamic acid (HCCA).

### Preparation of SP90-conjugated Liposomal Nanoparticles

A lipid film hydration method was used to prepare PEGylated liposomes composed of distearoylphosphatidylcholine, cholesterol, and PEG-DSPE, which were then used to encapsulate doxorubicin (Sigma-Aldrich) or to incorporate sulforhodamine B-DSPE (Avanti) [25], [36]. After preparation, the liposomes contained 110 to 130 µg doxorubicin per µmol phospholipid and had a particle size ranging from 65 to 75 nm in diameter. SP90-PEG-DSPE was subsequently incorporated into pre-formed liposomes by co-incubation at 60°C, the transition temperature of the lipid bilayer, for 1 hour with gentle shaking. After incubation, the surface of each liposome displayed about 500 peptide molecules. Sepharose 4B (GE Healthcare) gel filtration chromatography was used to remove released free drug, unconjugated peptides, and unincorporated conjugates. Doxorubicin concentrations in the fractions of eluent were determined by measuring fluorescence at λEx/Em = 485/590 nm using a spectrofluorometer (Spectra Max M5, Molecular Devices).

### MTT Cell Proliferation Assay

BT483 cells were seeded in 96-well plates (5000 cells/well) in DMEM media and were incubated overnight. After 24 hours, supernatants were removed and cells were treated with different concentration of drug in 2% FBS/DMEM for 72 hours. At the end of time point, 50 µl of MTT (Thiazolyl Blue Tetrazolium Bromide; Sigma-Aldrich) was added to each well of the plate and was incubated for 3.5 hours. After incubation, 150 µl of Dimethyl sulfoxide (DMSO; Mallinckrodt Baker) was added to each well for 10 min, and the absorbance was determined with microplate reader (SpectraMax M5, Molecular Devices) at 540 nm.

### Synthesis of SP90-conjugated Quantum Dots (SP90-QD)

Qdot 800 ITK amino PEG quantum dots (Invitrogen) were used in this study. The procedures for synthesis of ligand-conjugated QDs were described in a previous study [38]. Briefly, QDs were conjugated to sulfo-SMCC (Sulfosuccinimidyl-4-(*N*-maleimidomethyl) cyclohexane-1-carboxylate; Thermo Scientific) to generate a maleimide-activated surface on the QD, and free sulfo-SMCC was then removed using a NAP-5 desalting column (GE HealthCare). SP90 peptide were thiolated (addition of sulfhydryl) using Traut’s reagent (Thermo Scientific), and then purified with HPLC. Subsequently, the maleimide-functionalized QDs were incubated with SP90-SH at 4°C overnight. SP90-conjugated QDs were purified using a NAP-5 desalting column to remove any free peptides. The concentration of quantum dots was examined using a spectrofluorometer, and calculated through interpolation using a standard curve.

### Uptake of SP90-LD by Human Breast Cancer Cells

Tumor cells were grown on a 24-well plate to 90% confluency, and 2.5 µg/ml SP90-LD or LD in complete culture medium was added. The cells were incubated at 37°C, for the following periods of time: 1, 2, 4, 8, 16, 32 and 48 hours. At the indicated time point, cells were washed with PBS, and SP90-LD or LD on the cell surface was removed by adding 0.1 M Glycine, pH 2.8 for 10 min. Cells were then lysed with 200 µl 1% Triton X-100. For extraction of doxorubicin, 300 µl IPA (0.75 N HCl in isopropanol) was added to the lysate and shaken for 30 min. After the lysate was centrifuged at 12,000 rpm for 5 min, total doxorubicin was determined by measuring fluorescence at λ_Ex/Em_ = 485/590 nm using a spectrofluorometer (SpectraMax M5, Molecular Devices). The concentration of doxorubicin was calculated by interpolation using a standard curve.

### Animal Model for the Study of Ligand-targeted Therapy

Animal care was carried out according to guidelines established by Academia Sinica, Taiwan. The protocol was approved by the Committee on the Ethics of Animal Experiments of Academia Sinica (Permit Number: MMi-ZOOWH2009102). Mice 4–6 weeks old were injected subcutaneously in the dorsolateral flank with human breast cancer cells. Mice with size-matched tumors (approximately 75 mm^3^) were then randomly assigned to different treatment groups, and were injected with either liposomal doxorubicin (LD), SP90-conjugated liposomal doxorubicin (SP90-LD), free doxorubicin (FD) or equivalent volumes of saline through the tail vein. The dosage of doxorubicin was 3 mg/kg injected once a week for three weeks (on days 0, 7 and 14). Mice bearing large tumors (approximately 500 mm^3^) were treated with SP90-LD, LD or FD through tail vein injection, at a dose of 3 mg/kg twice a week, for a total dose of 9 mg/kg. Mouse body weight and tumor size were measured twice a week. At the end of the experiment, tumor tissue and the visceral organs of each mouse were removed and fixed with 3% formaldehyde, and OCT was embedded for further histopathological examination.

For the breast cancer orthotopic model, 1×10^7^ MCF-7-Luc (clones stably expressing luciferase) cells mixed with matrigel (BD) were orthotopically injected into the right inguinal mammary fat pad of each female SCID mouse. Once tumor size reached 500 mm^3^, the mice were treated with either SP90-LD, FD or LD by intravenous injection, with a total doxorubicin dosage of 9 mg/kg (3 mg/kg; twice a week for three injections). For measurement of tumor luciferase activity, the mice were injected intraperitoneally with luciferin (Caliper Life Sciences) and imaged using a Xenogen IVIS200 imaging system. Tumor size was monitored using a vernier caliper, and the tumor volume was calculated using the formula: length × (width)^ 2^ × 0.52.

### Endocytosis of SP90-conjugated Liposomes by BT483 Cells

BT483 cells were plated and grown to ∼80% confluence on coverslips. The cells were then incubated with either liposomal SRB (LS), SP90-Lipo-SRB (SP90-LS) or control peptide-Lipo-SRB (CP-LS) at 37°C in complete medium. After 1 hour of incubation, the cells were washed with PBS, stained with DAPI, and then examined using a Leica confocal microscope (TCS-SP5-AOBS). Images were processed by Leica Application Suite Advanced Fluorescence software.

To assay the liposomes in endosomes of BT483 cells, the cells were incubated with SP90-conjugated liposomal doxorubicin (SP90-LD) or control peptide-conjugated LD (CP-LD) at 37°C. After 5 min of incubation, the cells were washed with PBS, fixed using a high pressure freezer (EM PACT2, Leica), and then analyzed by transmission electron microscopy (CM100, Philips).

### Total White Blood Cell (WBC) Count

Three days after final treatment (on day 17), blood was extracted from the submaxillary vein and mixed gently with 15% EDTA solution to prevent coagulation. Red blood cell lysis buffer containing 2% acetic acid and 1% of Gentian violet (Sigma-Aldrich) was then added and incubated at room temperature. The total WBC was calculated using a hemacytometer.

### Terminal Deoxynucleotidyl Transferase–mediated dUTP Nick End Labeling (TUNEL) Staining

The frozen tumor tissue sections were incubated with terminal deoxynucleotidyl transferase-mediated dUTP nick end labeling reaction mixture (Roche Diagnostics) at 37°C for one hour. The slides were counterstained with mounting medium with DAPI (Vector Laboratories). The slides were then visualized under a fluorescent microscope and areas of TUNEL positive cells were quantified by pixel area count normalize with DAPI using MetaMorph software (Molecular Devices).

### Animal Model for Studying Tumor Localization of Liposomal Doxorubicin

SCID mice bearing breast cancer xenografts (∼300 mm^3^) were injected in the tail vein with either free drug doxorubicin (FD), targeting (SP90-LD) or non-targeting (CP-LD, and LD) liposomal doxorubicin, at a dose of 2 mg/kg. At 24 hours post-injection, three mice in each group were anaesthetized and sacrificed. The mice were perfused with 50 ml PBS to wash out doxorubicin in blood, and xenograft tumors were then removed and homogenized. Total doxorubicin was quantified by measuring fluorescence at λ_Ex/Em_ = 485/590 nm using a spectrofluorometer (SpectraMax M5, Molecular Devices).

To determine the presence of the drug in tumor tissues, frozen tumor sections were fixed and stained with DAPI. The fluorescence signal of doxorubicin was detected using an inverted fluorescence microscope (Axiovert, Zeiss) with a 546 nm excitation and 590 nm emission filter set.

### *In vivo* Imaging Analysis

Six 6-week old SCID female mice were subcutaneously implanted with 5×10^6^ BT483 cells. Mice with size-matched tumors (approximately 300 mm^3^) were then randomly divided into two groups and intravenously injected with 200 pmole of SP90-QD or QD. The mice were anesthetized using isofluoran, and the fluorescence images were captured using a Xenogen IVIS 200 imaging system (Excitation: 520/50 nm; Emission: 832/65 nm) at the indicated times. To quantitatively compare tumor accumulation of SP90-QD versus QD, the fluorescence intensity was calculated with background subtraction, using Living image software (Xenogen).

### Statistical Analysis

Student's *t*-test (unpaired and two-sided) was used to calculate *P* values. *P* < 0.05 was considered significant for all analyses. All values are represented as mean ± standard deviation (s.d.).

## Results

### Identification of Novel Peptides that Bind to Breast Cancer Cells

**Figure 1:**
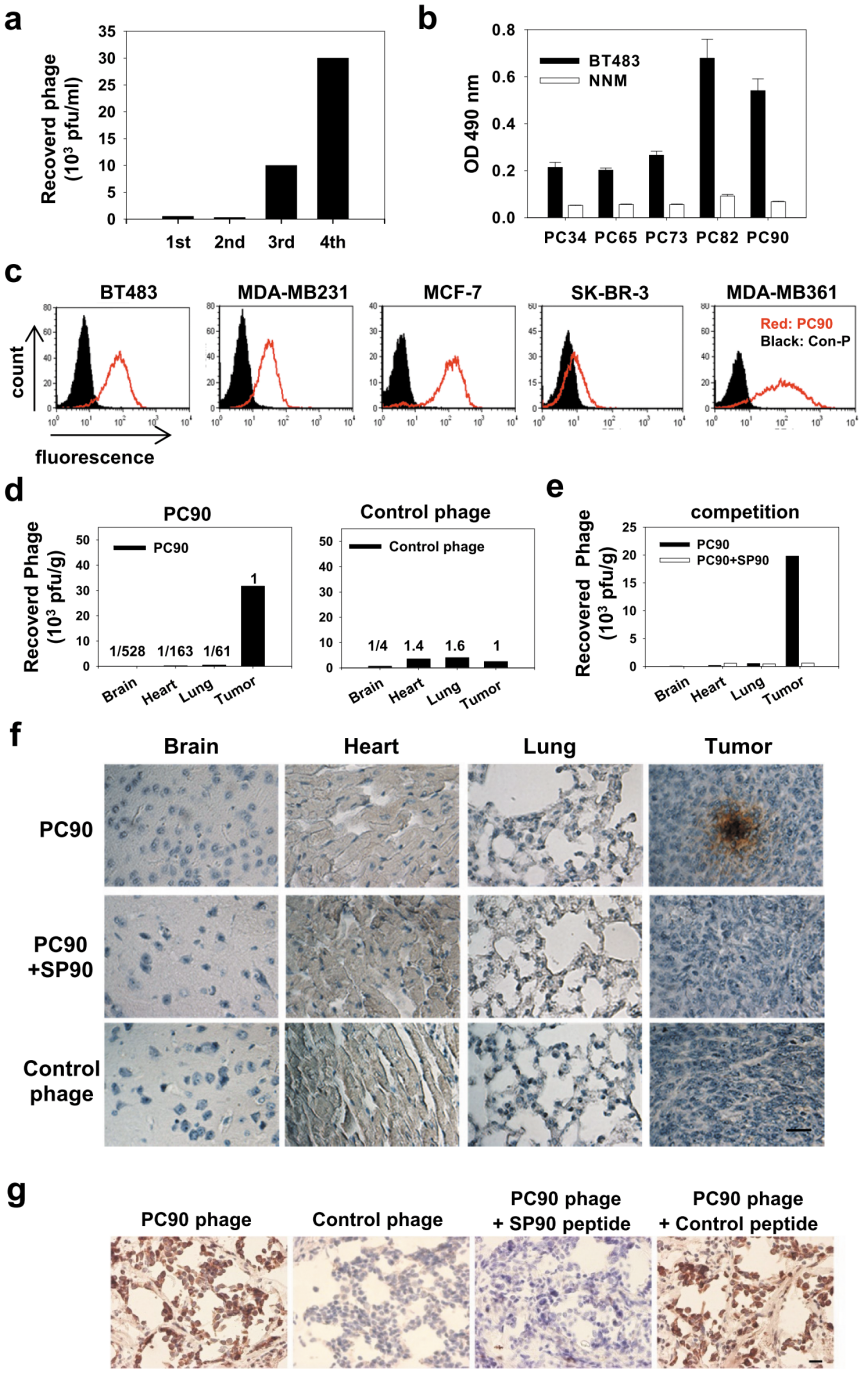
Identification of breast cancer cell-targeting peptides using *in vitro* phage display. **a**, A phage-display random peptide library was used to identify peptides that bind to the breast cancer cell line, BT483. After four rounds of biopanning, the titer of phage eluted from the breast cancer cells had increased 60-fold relative to the first round of selection. PFU, plaque-forming units. **b**, Binding activity and specificity between individual phage clones and breast cancer cells were tested by cellular ELISA. Bar, mean; Error bar, s.d.; *n* = 4; OD490 nm, optical density at 490 nm. **c**, The binding activity of the PC90 phage to five breast cancer cell lines was analyzed by flow cytometry. Con-P, control phage. **d**, Verification of tumor-homing ability of phages *in vivo*. SCID mice bearing human breast cancer xenografts received intravenous injections of PC90 and control phage. After perfusion with PBS buffer, xenograft tumor masses were removed and phage titers were measured. **e**, The tumor-homing ability of the PC90 phage was competitively inhibited by its cognate peptide SP90. **f**, Immunohistochemical localization of PC90 in figure 1d. Scale bar: 50 µm. **g**, Immunohistochemical staining of human surgical specimens of breast infiltrating ductal carcinoma using PC90 phage. Tumor sections of surgical specimens incubated with PC90 or control phage. The phage signal was detected using HRP-conjugated anti-M13 phage antibody. Scale bar: 20 µm.

In this study, we used a phage-displayed random peptide library to isolate phages that were able to bind to BT483 breast cancer cells. After four rounds of affinity selection (biopanning), the titer of bound phage increased by up to 60-fold (Fig. 1a). Through ELISA screening and DNA sequencing, we identified five phage clones (PC34, PC65, PC73, PC82 and PC90) with unique peptide sequences that bind to BT483, but not to control normal nasal mucosal (NNM) epithelial cells (Fig. 1b). The phage clones with higher BT483-binding activities; PC34, PC65, PC73, PC82 and PC90, displayed QNIYAGVPMISF, EATNSHGSRTMG, TVSWSTTGRIPL, QLEFYTQLAHLI and SMDPFLFQLLQL peptide sequence, respectively.

To verify whether these five phage clones would bind to target molecules expressed on the surface of breast cancer cells, the surface binding activity of each individual phage clone was analyzed by flow cytometry (Fig. S1a). The five phage clones exhibited prominent binding to BT483 cells, with PC90 displaying the best reactivity (Fig. S1a). We further demonstrated that PC90 did not bind to normal human mammary epithelial cells (HMEpiC) using flow cytometry analysis (Fig. S1b). Based on these findings, we chose to focus on PC90 for the rest of the study. To investigate whether PC90 may be effective against a broad spectrum of cancer cells, we used flow cytometry to analyze the binding ability of PC90 to five breast cancer cell lines (Fig. 1c). We found markedly positive shifts in fluorescence in BT483, MDA-MB-231, MCF-7 and MDA-MB-361, and a small shift in SK-BR-3 cells. These results indicate that the PC90 phage specifically binds to several breast cancer cell lines.

To investigate the targeting ability of the selected phage clones *in vivo*, we intravenously injected each clone into mice bearing BT483-derived tumor xenografts. After perfusion, we measured the phage titers in the tumor and normal organs [34], [39]. The results showed that the five phage clones (PC34, PC65, PC73, PC82 and PC90) but not control helper phages had tumor-homing ability (Fig.1d and Fig. S1c). Of these, PC90 targeted to tumors most efficiently, and was identified in tumor mass at concentrations ≧ 61-fold higher than that in the control organs. We subsequently synthesized the peptide displayed by the PC90 phage, SP90, which has the amino acid sequence SMDPFLFQLLQ. We found that co-administration of SP90 and PC90 into mice reduced recovery of PC90 from tumor masses by nearly 97%, suggesting that SP90 is able to competitively inhibit the binding of PC90 to breast cancer cells (Fig. 1e). We also examined the tissue distribution of PC90, by using anti-phage antibody to immunostain the tissue sections derived from homing experiments. PC90 was found to be selectively localized in tumor tissues rather than in normal tissues, whereas no immunoreactive product was found through control phage staining (Fig. 1f and Fig. S2). Moreover, when PC90 was co-injected with the synthetic peptide SP90, no immunoreactivity was found in the tumor tissue (Fig. 1f).

To determine whether this targeting ligand had affinity for human breast cancer surgical specimens, we performed immunohistochemistry staining with PC90 on breast infiltrating ductal carcinoma tissue sections. We found that PC90 could recognize the tumor cells of breast cancer surgical specimens, and that co-treatment with SP90 could competitively inhibit PC90 binding (Fig. 1g). Of the 20 breast cancer specimens from different patients, 90% (18/20) stained positive for PC90 (Table S1). These data indicate that PC90 can recognize unidentified molecules expressed on breast cancer cell lines, as well as on cells from the surgical specimens of breast cancer.

### SP90-conjugated Liposomes Exhibit Enhanced Drug Intracellular Delivery and Cytotoxicity

**Figure 2:**
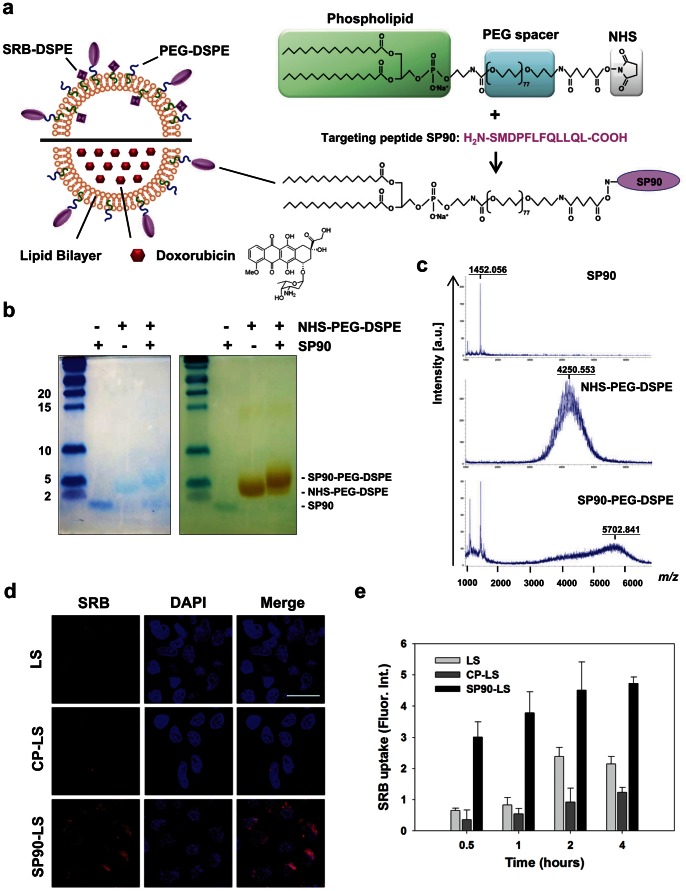
Generation of peptide-conjugated liposomal nanoparticles. **a,** Schematic illustration of the targeting liposome, depicting the lipid bilayer membrane encapsulating a large amount of doxorubicin or sulforhodamine B, and the breast cancer targeting ligands that can be displayed on the surface of the liposome. Targeting peptide SP90 was chemically conjugated to NHS-PEG-DSPE in a 1.1∶1 molar ratio. There were about 500 peptide molecules per liposome. **b**, The percentage of conjugation between SP90 and NHS-PEG-DSPE was confirmed by subjecting the reaction product to electrophoresis on a Tricine-SDS gel, staining with coomassie blue for peptide, followed by barium chloride for PEG (SP90∶1.4 kDa, NHS-PEG-DSPE: 4.3 kDa, SP90-PEG-DSPE: 5.7 kDa). PEGylation efficiency for SP90 was 85% based on quantification of band intensity by densitometry. **c**, The SP90-PEG-DSPE conjugate was analyzed by MALDI-TOF mass spectrometry (bottom panel). A major peak appears at *m*/*z* 5702.8, which can be assigned as the PEGylated SP90 conjugate. Unconjugated SP90 (split peak at *m*/*z* 1452) and unreacted PEG (a broad peak around *m*/*z* 4250) were also visualized in the mass spectrum, which correspond to the peaks in the top and middle panels, respectively. **d**, Internalization of SP90-liposomal SRB (SP90-LS) and nontargeted LS (CP-LS and LS) by BT483 cells was studied by confocal microscopy (SRB, red). Nuclear staining was by DAPI (blue) (Scale bar: 10 µm). **e**, Time-course of SRB uptake by BT483 cells treated with SP90-LS, CP-LS and LS at the indicated times.

To investigate whether SP90 could promote liposomal drug delivery in human breast cancer cells, SP90 was conjugated to NHS-PEG-DSPE. Upon insertion of the phospholipid DSPE, the PEGylated SP90 conjugates became coupled to the external surface of liposomal nanoparticles. These nanoparticles contained sulforhodamine B (SRB; fluorescence reagent) or doxorubicin (Fig. 2a). The PEGylation efficiency of SP90 was validated by Tricine-SDS-PAGE and MALDI-TOF MS (Fig. 2b and c).

The internalization ability of the targeting ligand is an essential property for successful tumor-targeted liposomal drug delivery. As such, we examined internalization of SP90-conjugated liposomal SRB (SP90-LS) in tumor cells using confocal microscopy. We observed a large amount of SRB in the cytoplasm of BT483 cells incubated with SP90-LS at 37°C, whereas little SRB fluorescence was detectable in cells incubated with non-targeting liposomal SRB (LS) or control peptide-conjugated liposomal SRB (CP-LS) (Fig. 2d). This indicates that conjugation of the liposome with SP90 resulted in effective internalization of SRB. Furthermore, we found that SP90 markedly enhanced intracellular SRB uptake by cancer cells at each time point examined (Fig. 2e).

**Figure 3:**
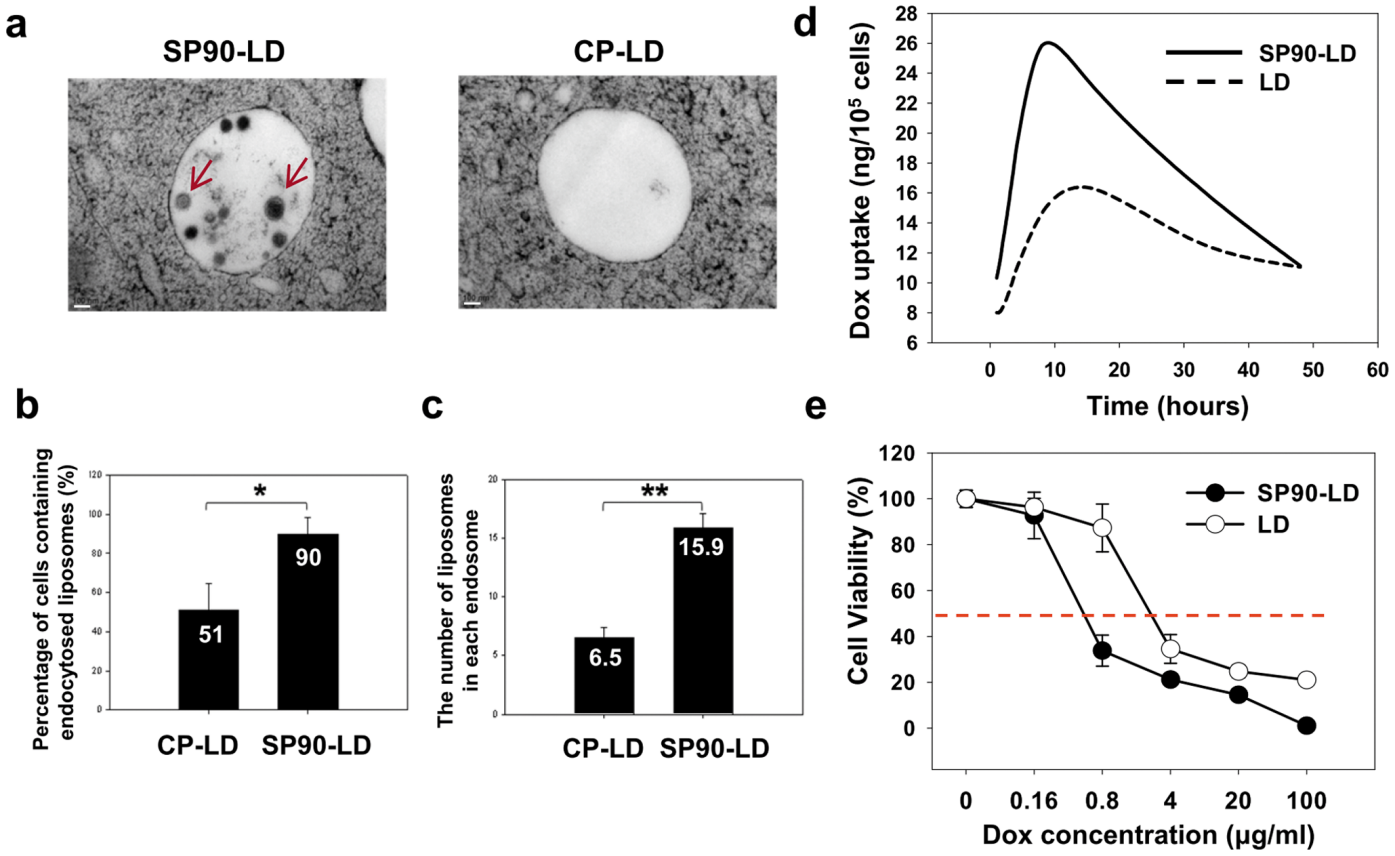
SP90-conjugated liposomes enhanced drug delivery and cytotoxicity towards cancer cells, through increased endocytosis. **a,** Electron micrographs revealing liposomes in the endosomes of BT483 cells treated with SP90-LD (arrows) or CP-LD. Scale bar: 100 nm. **b,** The percentage of breast cancer cells harboring endocytosed liposomes following treatment with SP90-LD or CP-LD (*n* = 50 in each group; *P<0.01). **c,** The average number of liposomes in each endosome following treatment with SP90-LD or CP-LD (n = 20 in each group; **P<0.01). **d,** Cells were incubated with either SP90-LD or LD at 37°C. Doxorubicin uptake by the cells was quantified at the indicated times, following the removal of surface-bound liposomal drugs. **e,** Cells were treated with SP90-LD and LD at varying concentrations. Cell viability was determined by MTT assay, and calculated as a percentage of living cells. Each point represents the mean of four experiments. Error bar, s.d.

To verify internalization of SP90-conjugated liposomes through receptor-mediated endocytosis, we used transmission electron microscopy (TEM) to analyze the endosomes of tumor cells treated with either SP90-conjugated liposomal doxorubicin (SP90-LD) or control peptide-conjugated liposomal doxorubicin (CP-LD) at 37°C for 5 minutes. As shown in Figure 3a, SP90-LD accumulated in the endosomes of cancer cells to a much greater extent than CP-LD. Endocytosed liposomes were observed in 90% of cells treated with SP90-LD, but only in 51% of cells treated with CP-LD (Fig. 3b). The average number of liposomes observed in each endosome was 2.4 fold higher in cells treated with SP90-LD as compared to cells treated with CP-LD (Fig. 3c).

Doxorubicin is a small-molecule chemotherapeutic agent that is historically important in the treatment of breast cancer. To identify whether SP90-conjugated liposomes may enhance delivery of doxorubicin, we treated BT483 cells with equal concentrations of LD or SP90-LD. The uptake of doxorubicin was quantitatively measured, based on fluorescent intensities at several time points (Fig. 3d). Cellular uptake of doxorubicin was elevated in BT483 cells through treatment with SP90-LD. The area under the concentration-time curve (AUC_0–48_ hour) was 2.36-fold larger for SP90-LD as compared to LD in breast cancer cells (Table S2). To assess whether SP90-conjugation enhanced the therapeutic potential of LD, we performed *in vitro* cytotoxicity assays for SP90-LD in BT483 cells. Compared with LD, SP90-LD significantly reduced the viability of cancer cells, and promoted a 4.9-fold decrease of the half maximal inhibitory concentration (*IC_50_*) in BT483 cells (Fig. 3e and Table S2). However, enhancement of the cytotoxic effect of SP90-LD was subject to competitive inhibition upon co-treatment with free SP90 peptides, in a dose-dependent manner (Fig. S3a). The cell viability of BT483 cells was not affected by treatment with SP90 peptides alone (Fig. S3b).

### Therapeutic Efficacy of SP90-mediated Drug Delivery System in Mouse Models

**Figure 4:**
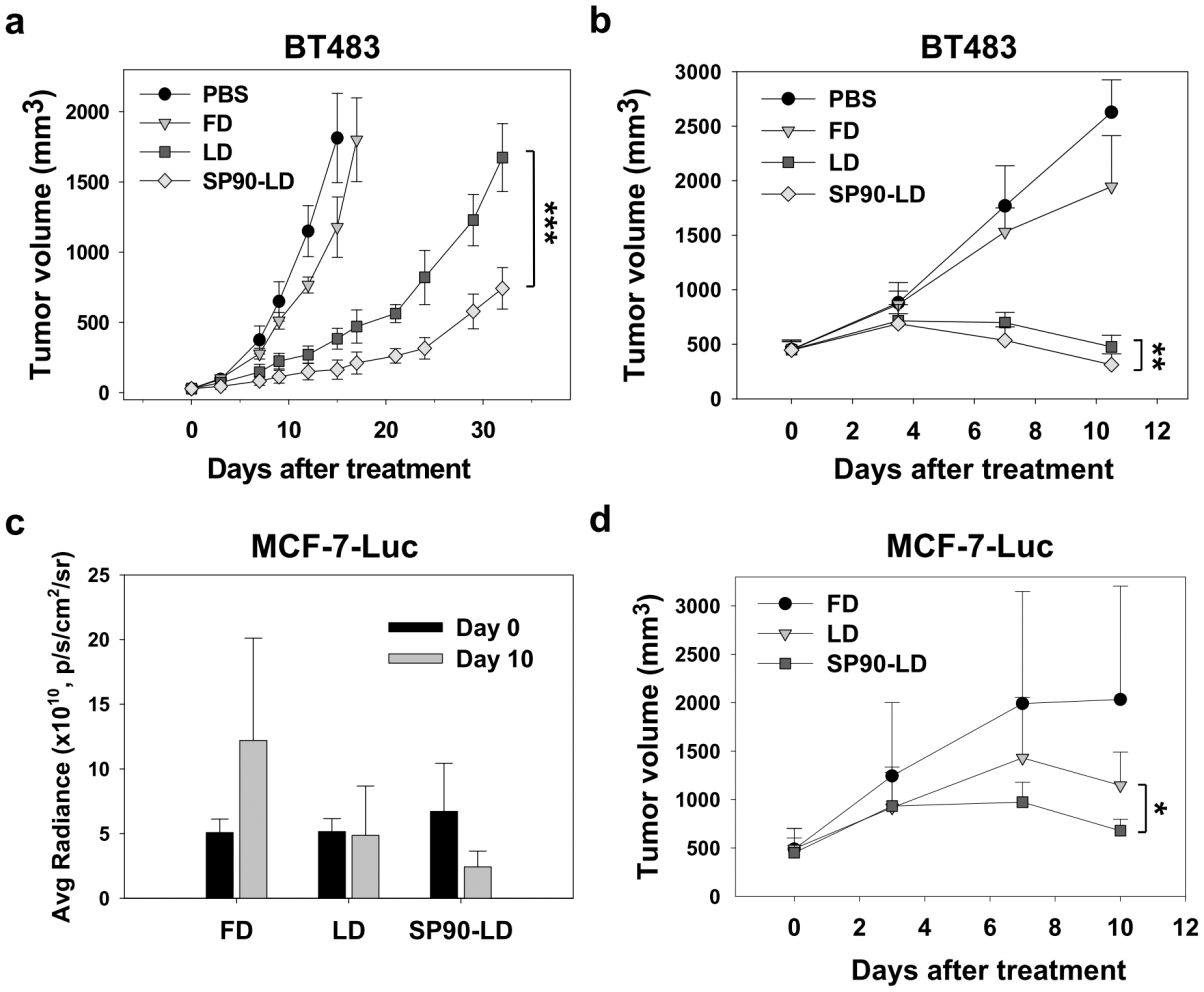
Treatment of SCID mice bearing human breast cancer xenografts with SP90-LD. **a**, Mice bearing BT483-derived breast cancer xenografts with average tumor size of ∼75 mm^3^ were treated with 3 mg/kg at weekly intervals (for a total of three injections) of either FD, LD, SP90-LD, or an equal volume of PBS by intravenous injection. *n* = 6 each group. Points, mean tumor volumes. **b**, Mice bearing size-matched BT483-derived breast cancer xenografts with tumor size of ∼500 mm^3^ were treated with SP90-LD, LD, or FD, for a total doxorubicin dosage of 9 mg/kg (3 mg/kg/injection, twice a week). *n* = 8. The average tumor volumes at cessation of treatment in the LD, FD and control PBS groups were 1.5, 6.2 and 8.4 times larger than that in the SP90-LD-treated group, respectively. **c**, Mice bearing orthotopic breast cancer tumors with average tumor size of ∼500 mm^3^, MCF-7-Luc, were injected with either SP90-LD, FD or LD, for a total doxorubicin dosage of 9 mg/kg (3 mg/kg/injection, twice a week). The tumors were imaged and luminescence was quantified at the indicated days by IVIS200. *n* = 5. **d**, Volume of tumors depicted in (c). Error bar, s.d.; *P<0.05; **P<0.01; ***P<0.001.

To evaluate the potential of SP90 in improving the efficacy of anticancer chemotherapy *in vivo*, we formulated a targeted drug delivery system by coupling SP90 with PEGylated liposomal doxorubicin (SP90-LD). SCID mice bearing BT483-derived xenografts (∼75 mm^3^) were treated with SP90-LD, liposomal doxorubicin (LD), free doxorubicin (FD) or equivalent volumes of PBS. All formulations were injected intravenously at a total doxorubicin dosage of 9 mg/kg (3 mg/kg at weekly intervals). Anticancer efficacy was evaluated by determining the average tumor volume throughout the 32 days treatment period. The tumors in mice administered with SP90-LD were found to be 2.3-fold smaller in volume than those administered with LD alone on day 32 (Fig. 4a). To evaluate the side-effects caused by the systemic delivery of chemotherapeutic drugs, we measured total white blood cell (WBC) count and body weight. The average total WBC count of the SP90-LD group (4.8×10^3^/mm^3^) was similar to that of the PBS (5.0×10^3^/mm^3^) and FD (5.6×10^3^/mm^3^) groups, but higher than that of the LD (2.6×10^3^/mm^3^) group (Fig. S4a). Administration of SP90-LD did not cause an appreciable reduction in body weight as compared to the LD group (Fig. S4b and c).

To verify whether SP90-LD treatment would be effective against human breast tumor xenografts with a greater volume, mice bearing large BT483-derived xenografts (500 mm^3^) were intravenously administered with one of three drug formulations at a total doxorubicin dosage of 9 mg/kg (3 mg/kg twice a week). By the cessation of treatment, tumors treated with SP90-LD were significantly smaller than those treated with non-targeting LD (Fig. 4b). The histopathology of tumor tissues in each treatment group was subsequently examined by H&E staining. Markedly disseminated necrotic/apoptotic areas were presented throughout the whole section of SP90-LD-treated xenografts, while fewer necrotic/apoptotic areas were present in the tumors of the LD-treated group (Fig. S5). We further carried out TUNEL assay to examine apoptotic cells in tumor region. The number of apoptotic cells in the SP90-LD-treated group was significantly greater (2.5-fold) than in the LD-treated group (Fig. S6).

Orthotopic tumor models are more pertinent with respect to both host-tumor interactions and response to therapy. Therefore, we established an orthotopic mouse model of breast tumor by implanting MCF-7 cells stably expressing luciferase (MCF-7-Luc) into murine mammary pads, enabling us to further elucidate the therapeutic response of SP90-targeting liposomes. Once tumor size reached 500 mm^3^, mice were treated with either SP90-LD, LD or FD. Tumor growth was monitored using luminescent imaging and a vernier caliper twice a week. By the cessation of treatment (day 10), the level of bioluminescence in the tumors of SP90-LD-treated mice was lower than that of FD- and LD-treated mice (by 8- and 2-fold, respectively) (Fig. 4c and S7a). Similarly, the average tumor volume and weight in the SP90-LD-treated group was significantly lower compared to that of the FD- and LD- treated groups (Fig. 4d and S7b).

### SP90-conjugated Liposomes Improved Drug Delivery *in vivo*

**Figure 5:**
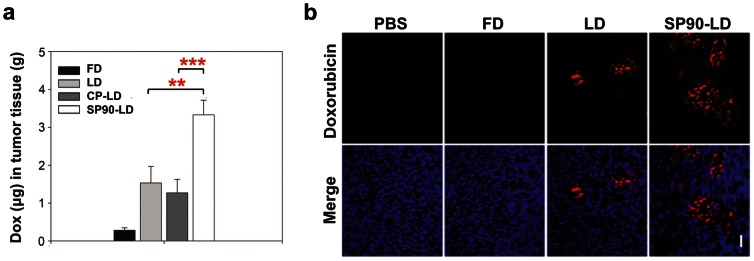
SP90-conjugated liposomes enhanced drug delivery to tumor. **a,** BT483 breast cancer-bearing mice were treated with different formulations of liposomal (LD, CP-LD and SP90-LD) and free doxorubicin (FD). After perfusion with 50 ml PBS, doxorubicin concentration was determined in tumor tissue (*n* = 3 in each group; **P<0.01, ***P<0.005). **b**, Representative two-color images showing the distribution of doxorubicin (red) in relation to nuclei (blue) in tumor tissue sections. Accumulation of doxorubicin in tumor nuclei was examined at day 3 post-injection. Scale bar, 50 µm. Bar, mean; Error bar, s.d.

To explore the mechanisms underlying the enhanced inhibitory effects using SP90-conjugated liposomal drugs, we examined drug accumulation in tumor tissues by injecting SCID mice bearing BT483 xenografts with FD, LD, control peptide-conjugated LD (CP-LD) or SP90-LD. The mean intra-tumor doxorubicin concentration in the SP90-LD group was 12.0-, 2.2- and 2.6-fold higher than that in the FD, LD and CP-LD groups, respectively (Fig. 5a). As it is possible that extensive perfusion caused LD to be washed out of the tumor tissues, we repeated this experiment without PBS perfusion. Accumulation of SP90-LD in tumors continued to be higher than that of FD (9.4-fold) and non-conjugated LD (1.5-fold) (Fig. S8). To visualize the drug delivery profile of the three doxorubicin formulations, we examined intracellular uptake of doxorubicin in tumor tissues by fluorescence microscopy. We found areas with detectable doxorubicin to be larger in the nucleus of SP90-LD-treated tumors than in LD-treated tumors, while no detectable doxorubicin was found in FD-treated tumors (Fig. 5b). These experiments demonstrate that SP90 can elevate delivery and penetration of anticancer drugs into the tumor, resulting in accumulation of the drug at its intracellular target site, and thereby enhancing its therapeutic effect.

### *In vivo* Imaging of SP90-conjugated Nanoparticles

**Figure 6:**
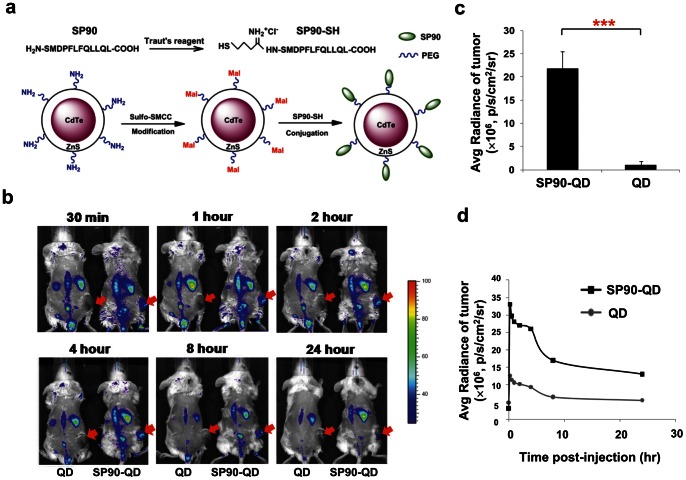
*In vivo* tumor targeting and imaging with SP90-conjugated quantum dots. **a**, SP90 was thiolated using Traut’s reagent to generate thiol-modified SP90 (SP90-SH). SP90-SH was subsequently conjugated with sulfo-SMCC-activated QD to produce SP90-QD. Mal, Maleimide. **b**, *In vivo* fluorescence imaging of SCID mice bearing BT483-derived tumors was performed after intravenous injection of QD (left) or SP90-QD (right). *n* = 3. Red arrows indicate the tumor loci. The NIR fluorescence images were captured at the indicated time points. **c**, Fluorescence signals within tumors were quantified using IVIS200 software. *n* = 3; Error bar, s.d; ***P<0.001. **d**, Quantification and kinetics of *in vivo* targeting of SP90-QD. Fluorescence intensity was recorded as photons per second per square centimeter per steradian (p/s/cm^2^/sr). Representative images from three independent sets of studies all gave similar results. *n* = 3.

To investigate whether SP90 could be used to enhance imaging of tumors, we constructed SP90-conjugated quantum dots (SP90-QD; Fig. 6a), and used flow cytometry to confirm their tumor-binding activity (Fig. S9). BT483-xenograft mice were then intravenously injected with QD and SP90-QD. At 30 minutes post-injection, we detected a higher near-infrared (NIR) fluorescence signal in the tumor sites of SP90-QD-treated mice than those in QD-treated mice (Fig. 6b). The greatest contrast between the fluorescence intensity of tumors in SP90-QD versus QD occurred at 2 hours post-injection, with a ratio of 22∶1 (Fig. 6c). Kinetic evaluation of imaging over 24 hours further confirmed that SP90-QD was both efficiently targeted to, and retained in tumors (Fig. 6d). These results indicate that the SP90-mediated drug delivery systems show great promise for their applications in tumor-targeted drug delivery and imaging.

## Discussion

Earlier diagnosis and better targeted drug delivery would significantly improve the efficacy of cancer therapy. Identification of tumor-targeting agents is instrumental in achieving these objectives. One promising approach to treating cancer is the use of ligand-conjugated liposome-encapsulated drugs that target tumor cells and blood vessels. Peptides that specifically bind to tumor targets can be coupled to the PEG terminus of sterically stabilized liposomes to precisely deliver chemotherapeutic agents to tumor cells. In this study, we searched for specific peptide ligands that would target breast cancer specifically, in order to develop a ligand-mediated target therapy capable of treating this disease.

The peptide-mediated targeting liposomes designed in this study offer several advantages over the use of free drugs in treatment of breast cancer. First, each peptide-conjugated liposome can efficiently deliver 15,000 anticancer drug molecules directly into endosome compartments with low pH; the liposomes then break down and release the encapsulated drug into the intracellular space of the target tumor cells [40], enabling controlled drug release and improving drug bioavailability (Fig. 3 and 5). Second, targeted and sustained release of the drug molecules from encapsulated carriers can increase therapeutic index of these chemotherapeutic agents against the tumor, while simultaneously reducing the toxicity of these drugs on the normal tissues (Fig. 4); importantly, conjugation to SP90 significantly reduced the toxicity of liposomal doxorubicin to white blood cells (Fig. S2), one of the major adverse effects of this drug in the clinical setting [41]. Third, enhanced drug accumulation in tumor tissues may help circumvent the problems associated with delivering drugs to solid tumors with high interstitial fluid pressure (Fig. 5) [5], [42], [43]. Our results revealed that SP90-LD more severely damaged blood vessels (data not shown) and cancer cells in tumor tissue as compared to LD in therapeutic experiments (Fig. S5 and S6). However, we have no evidence to suggest that SP90 can bind vascular endothelial cells, and hence tumor vessels were most likely killed indirectly, as a result of the diffusion of small-molecule drugs from targeted cells to neighboring cells through the bystander effect.

Although monoclonal antibodies have shown clinical benefits as anti-tumor agents, their potential for use as a drug delivery system has been limited due to a number of factors, including large molecular size, poor tumor penetration, and high immunogenicity when used in immunoliposomes [44]. Additionally, antibody-based drug delivery may lead to higher than normal levels of toxicity in liver and bone marrow, due to nonspecific antibody uptake by Fc receptor-expressing normal cells [44]. These limitations can be overcome by using peptide ligands, which are smaller, less immunogenic, and more cost-effective to produce and manipulate [34], [39]. Furthermore, multivalent peptide ligands have only a moderate affinity to tumor antigens, which is potentially advantageous for targeted drug delivery [26], since the strong affinity of antibodies may limit the penetration depth of their cargo into tumors [45], [46].

We found that most surgical specimens from breast cancer patients could be detected by SP90-bearing phage, PC90 (Fig. 1 and Table S1), further supporting the potential clinical application of this novel peptide ligand. Conjugating pharmaceutical nanocarriers or tumor imaging agents with SP90 may improve the effectiveness of current chemotherapeutic and diagnostic options for human breast cancer, by increasing their sensitivity and specificity. To explore whether the putative receptor can recognize a peptide ligand with sequence of SP90, we performed Alignment Search for this 12-mer peptide using BLASTP program [47]. No human protein sequences or conserved domains were found to be homologous to SP90 based on our results. However, the targeted cell surface molecule recognized by SP90 needs to be identified, in order to elucidate the mechanism of action of SP90 binding and to address safety concerns prior to clinical trials.

In conclusion, we identified several novel peptides, including SP90, capable of binding specifically to the cell surface of breast cancer cells both *in vitro* and *in vivo*. Linking SP90 to liposomes containing doxorubicin increased the therapeutic efficacy in mice with human breast cancer xenografts, through enhanced tumor apoptosis and decreased tumor angiogenesis. Quantification and visualization of doxorubicin levels also revealed increased drug concentrations in tumor tissues targeted by the liposome, highlighting the enhancement in both delivery and penetration of doxorubicin into the tumor. Our results indicate that the SP90 peptide may be used to enable specific targeting of tumor cells in the treatment of breast cancer, as well as to facilitate diagnosis of this malignancy.

## Supporting Information

Figure S1 **Verification of binding and** ***in vivo*** **tumor-homing ability of phages. a,** The surface binding activity of each selected phage to breast cancer and NNM cells was determined by flow cytometry. **b**, The binding activity of PC90 phage to normal human mammary epithelial cells (HMEpiC) was determined by flow cytometry. BT483 cells were used as positive. **c,** SCID mice bearing a BT483 xenograft tumor received intravenously injections of PC34, PC65, PC73, PC82, and control helper phage. After perfusion with PBS buffer, xenograft tumor masses and organs were removed and phage titers were measured. Phage titer in control organs are compared with tumor tissues, as indicated.
(JPG)

Figure S2 **The low-magnification images of PC90 immunohistochemical staining in tumor-homing analysis (** **Fig. 1e** **).** The PC90 phage was localized on tumor tissues and no localization was observed in normal organs such as the brain, heart, and lungs. Neither tumor cells nor normal organs were found to have immunoreactivity with control phage.
(JPG)

Figure S3 **Competition analysis of SP90-LD-induced cytotoxic effect by free SP90 peptides. a,** BT483 cells were treated with various concentrations of LD or SP90-LD in the presence of 10, 1, 0.1 or 0 µg/ml of SP90 peptides. **b,** BT483 cells were incubated with free SP90 and control peptides at various concentrations. After incubation for three days, cell viability was determined by MTT assay, and was calculated as a percentage of living cells. Each point represents the mean of three experiments. Error bar, s.d.
(JPG)

Figure S4 **Response of SCID mice bearing BT483-derived xenografts to the administration of SP90-LD in** **Figure 3a** **. a**, The effect of different treatments on white blood cell (WBC) counts. SP90-LD reduced the WBC toxicity of liposomal doxorubicin in the breast cancer xenograft model (*n* = 6 in each group; ** *P*<0.001). **b**, The body weight of each group. **c**, The effect of different treatments on change in body weight during the period from day 0 to day 20 (*n* = 6 in each group).
(JPG)

Figure S5 **Histopathological examination of SP90-LD-treated breast cancer xenografts.** After cessation of treatment, PBS- and FD-treated tumors were removed on day 20, while LD- and SP90-LD-treated tumors were removed on day 32 for histopathological examination. **a**, Tumor tissues were examined after staining with H&E. Markedly disseminated necrotic/apoptotic areas were observed throughout the entire section of SP90-LD-treated xenografts. LD-treated xenografts presented with moderate necrotic/apoptotic areas, while normal breast cancer cells were observed in the FD- and PBS-treated groups. (Scale bar, 100 µm). **b**, The percentage areas of necrosis/apoptosis were determined (*n = *6) at low magnification. The average percentage area of necrosis/apoptosis was markedly increased in the SP90-LD treated group as compared to the LD-, FD- or PBS-treated groups (*n* = 6, **P<0.01).
(JPG)

Figure S6 **SP90-conjugated targeting liposomes increased therapeutic efficacy through enhanced cancer cell apoptosis. a**, Sections were TUNEL-labeled to visualize apoptotic tumor cells (green). TUNEL-positive tumor cells were distributed more extensively in the SP90-LD-treated groups than in the LD, FD or PBS groups. **b**, Areas of TUNEL positive cells were quantified by pixel area count, and normalized to DAPI using MetaMorph Software. A significantly greater average apoptotic area was observed for the xenografts of the SP90-LD-treated group, as compared to those of the LD, FD or PBS treated groups. Scale bar, 85 µm.
(JPG)

Figure S7 **Treatment of SCID mice with SP90-LD in orthotopic human breast models. a**, Representative images used for the analysis described in Figure 4c. Luminescent radiance was assessed by IVIS200 imaging on the indicated days. *n* = 5. **b**, At the end of treatment, mice were sacrificed, and tumors were dissected and weighed. *P<0.05.
(JPG)

Figure S8 **SP90-conjugated liposomes enhanced drug delivery to tumor.** Accumulation of doxorubicin in tumors of breast cancer-bearing mice treated with different formulations of liposomal and free doxorubicin, without PBS perfusion (*n* = 3 in each group; *P<0.05).
(JPG)

Figure S9 **Analysis of tumor binding activity of SP90-QDs** ***in vitro*** **.** The binding activity of QD-labeled SP90 to BT483 cells was analyzed by flow cytometry.
(JPG)

Table S1 **Detection of human breast cancer surgical specimens by PC90 phages using immunohistochemistry.**
(DOCX)

Table S2 **AUC and IC_50_ of BT483 treated with SP90-LD and LD.**
(DOCX)
