# Supplementary figures and images for: Targeted Drug Delivery Systems Mediated by a Novel Peptide in Breast Cancer Therapy and Imaging
Source: PLoS One. 2013 Jun 11;8(6):e66128. doi: 10.1371/journal.pone.0066128 (PMC3679013; doi:10.1371/journal.pone.0066128)

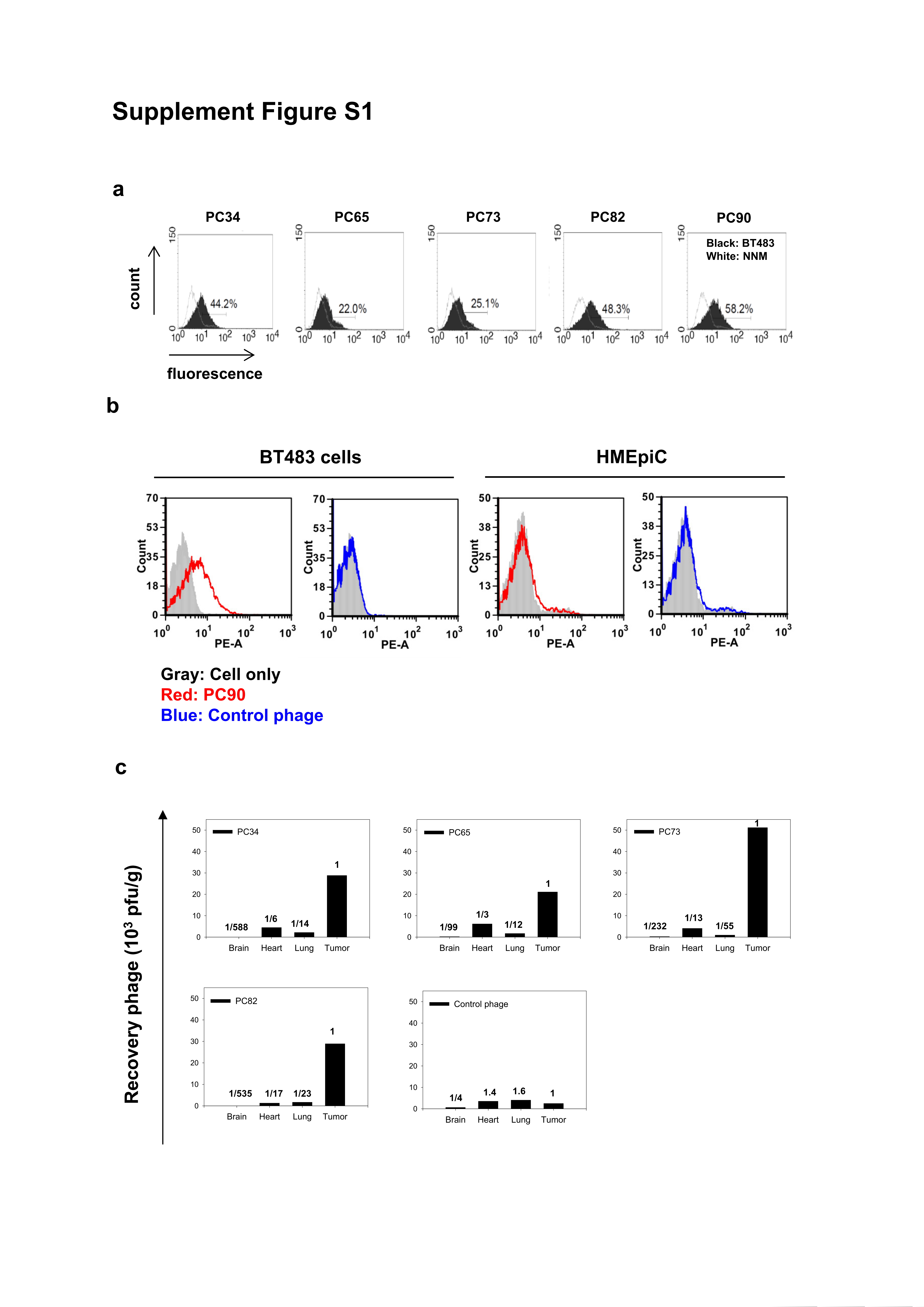

Supplement: Figure S1 — Verification of binding and in vivo tumor-homing ability of phages. a, The surface binding activity of each selected phage to breast cancer and NNM cells was determined by flow cytometry. b, The binding activity of PC90 phage to normal human mammary epithelial cells (HMEpiC) was determined by flow cytometry. BT483 cells were used as positive. c, SCID mice bearing a BT483 xenograft tumor received intravenously injections of PC34, PC65, PC73, PC82, and control helper phage. After perfusion with PBS buffer, xenograft tumor masses and organs were removed and phage titers were measured. Phage titer in control organs are compared with tumor tissues, as indicated. (JPG) [file pone.0066128.s001.jpg]

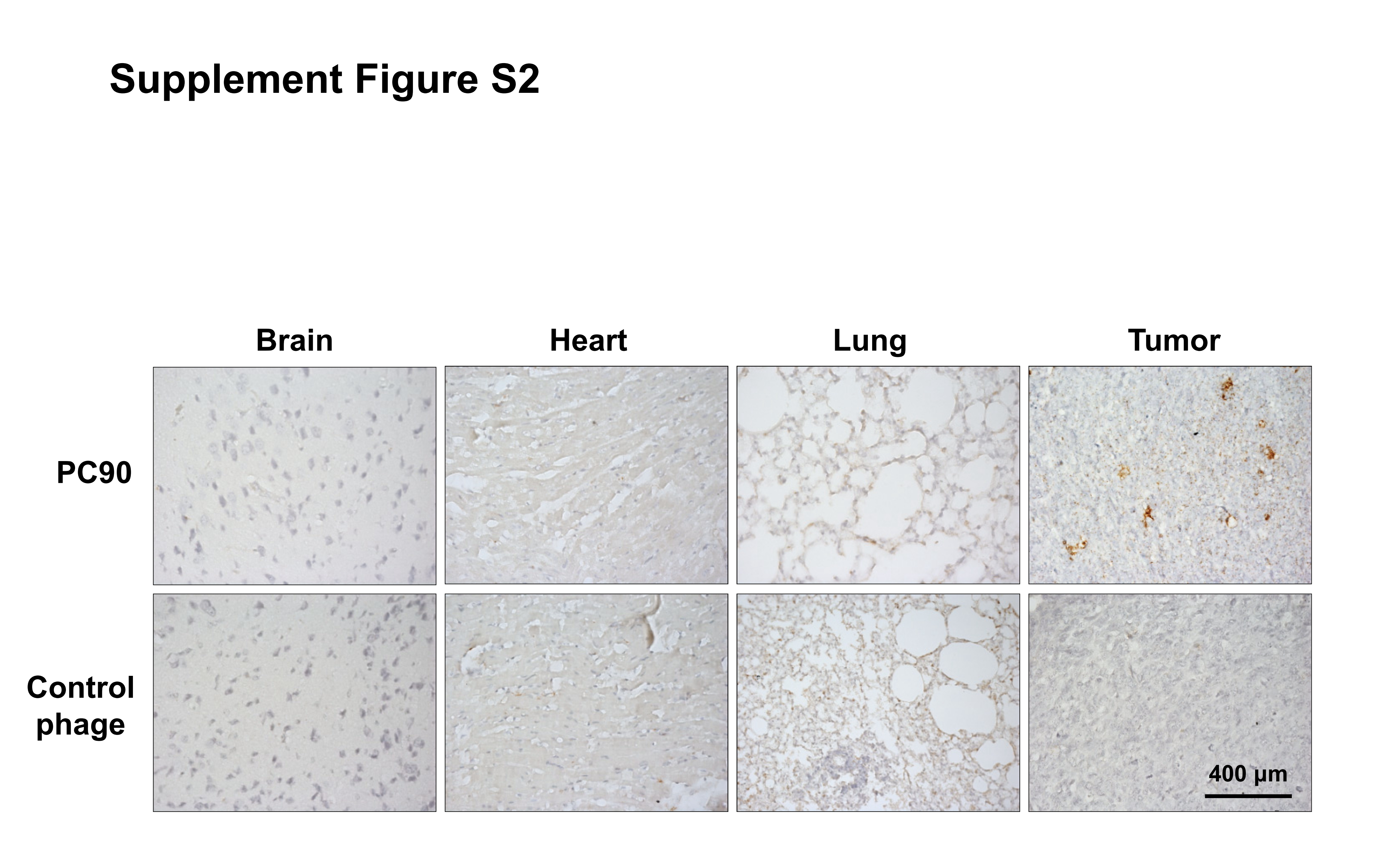

Supplement: Figure S2 — The low-magnification images of PC90 immunohistochemical staining in tumor-homing analysis ( Fig. 1e ). The PC90 phage was localized on tumor tissues and no localization was observed in normal organs such as the brain, heart, and lungs. Neither tumor cells nor normal organs were found to have immunoreactivity with control phage. (JPG) [file pone.0066128.s002.jpg]

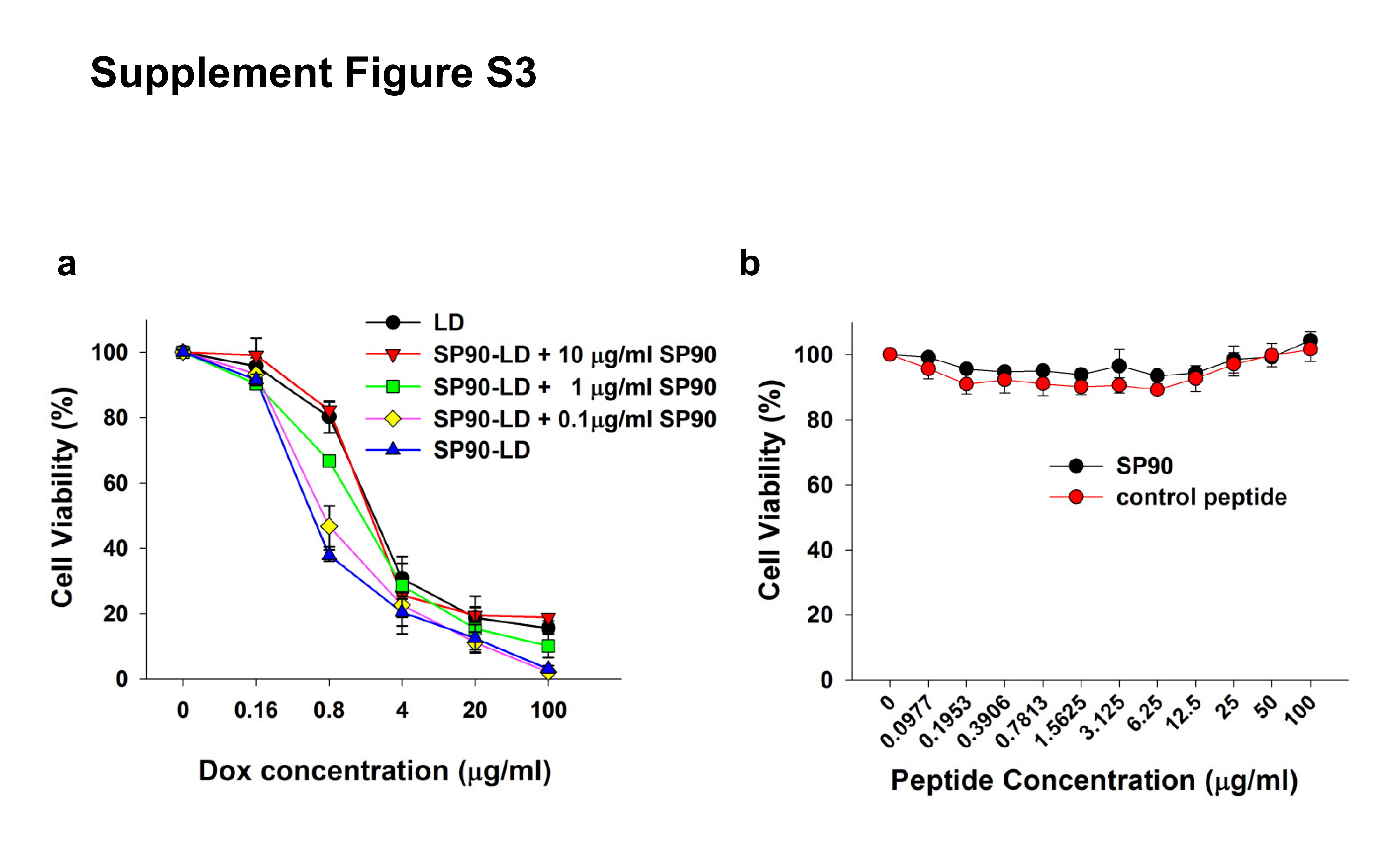

Supplement: Figure S3 — Competition analysis of SP90-LD-induced cytotoxic effect by free SP90 peptides. a, BT483 cells were treated with various concentrations of LD or SP90-LD in the presence of 10, 1, 0.1 or 0 µg/ml of SP90 peptides. b, BT483 cells were incubated with free SP90 and control peptides at various concentrations. After incubation for three days, cell viability was determined by MTT assay, and was calculated as a percentage of living cells. Each point represents the mean of three experiments. Error bar, s.d. (JPG) [file pone.0066128.s003.jpg]

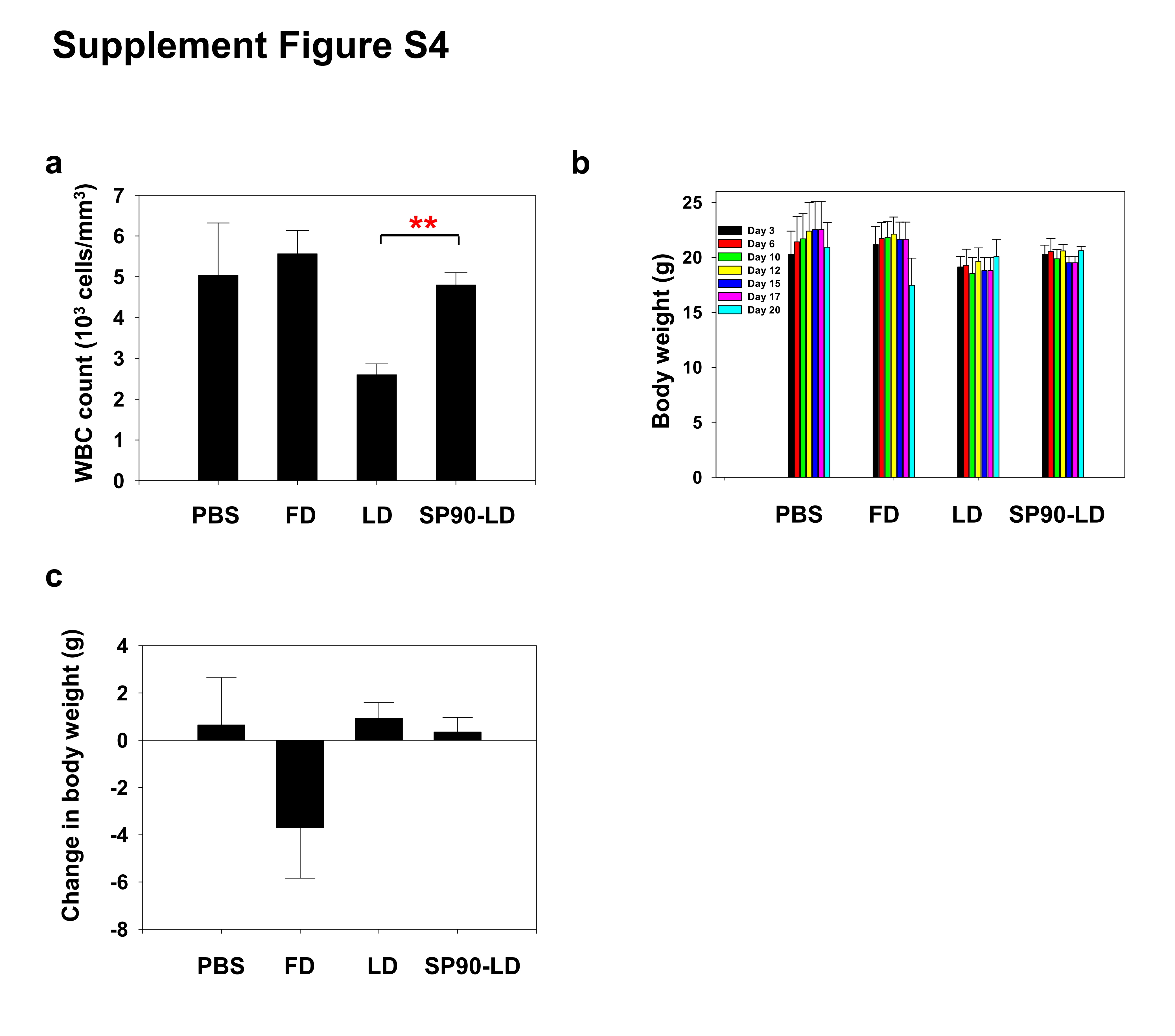

Supplement: Figure S4 — Response of SCID mice bearing BT483-derived xenografts to the administration of SP90-LD in Figure 3a . a, The effect of different treatments on white blood cell (WBC) counts. SP90-LD reduced the WBC toxicity of liposomal doxorubicin in the breast cancer xenograft model (n = 6 in each group; ** P<0.001). b, The body weight of each group. c, The effect of different treatments on change in body weight during the period from day 0 to day 20 (n = 6 in each group). (JPG) [file pone.0066128.s004.jpg]

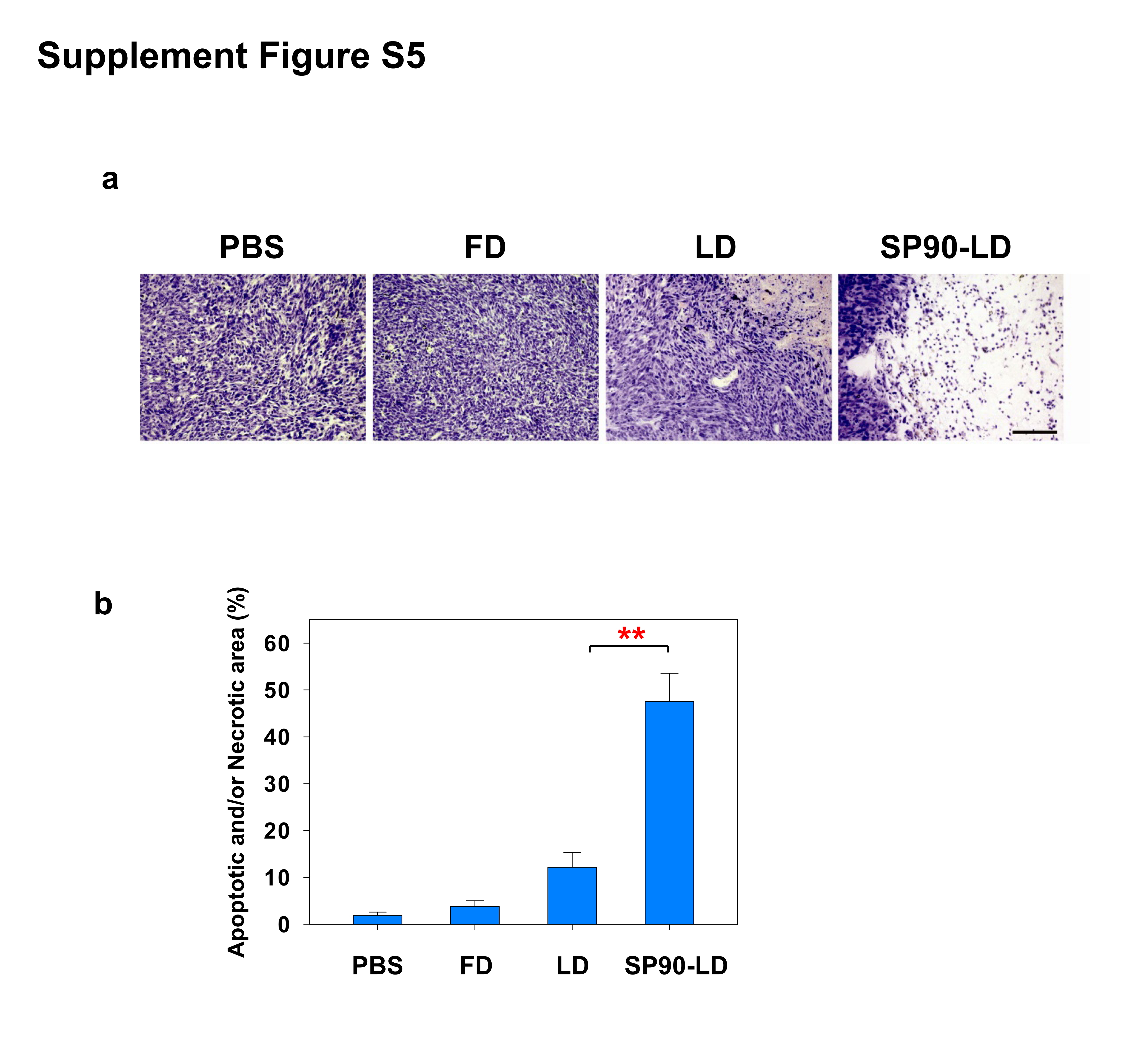

Supplement: Figure S5 — Histopathological examination of SP90-LD-treated breast cancer xenografts. After cessation of treatment, PBS- and FD-treated tumors were removed on day 20, while LD- and SP90-LD-treated tumors were removed on day 32 for histopathological examination. a, Tumor tissues were examined after staining with H&E. Markedly disseminated necrotic/apoptotic areas were observed throughout the entire section of SP90-LD-treated xenografts. LD-treated xenografts presented with moderate necrotic/apoptotic areas, while normal breast cancer cells were observed in the FD- and PBS-treated groups. (Scale bar, 100 µm). b, The percentage areas of necrosis/apoptosis were determined (n = 6) at low magnification. The average percentage area of necrosis/apoptosis was markedly increased in the SP90-LD treated group as compared to the LD-, FD- or PBS-treated groups (n = 6, **P<0.01). (JPG) [file pone.0066128.s005.jpg]

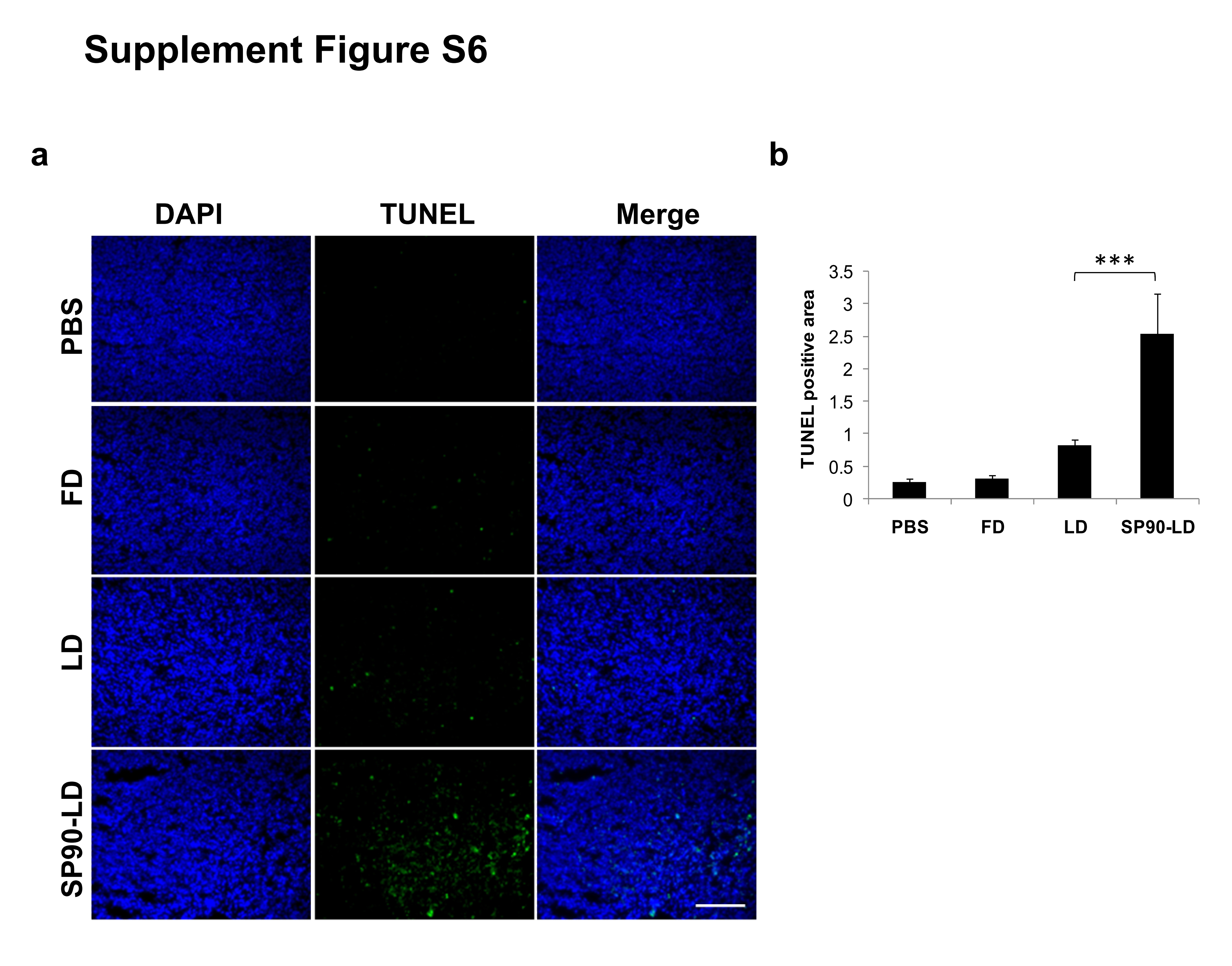

Supplement: Figure S6 — SP90-conjugated targeting liposomes increased therapeutic efficacy through enhanced cancer cell apoptosis. a, Sections were TUNEL-labeled to visualize apoptotic tumor cells (green). TUNEL-positive tumor cells were distributed more extensively in the SP90-LD-treated groups than in the LD, FD or PBS groups. b, Areas of TUNEL positive cells were quantified by pixel area count, and normalized to DAPI using MetaMorph Software. A significantly greater average apoptotic area was observed for the xenografts of the SP90-LD-treated group, as compared to those of the LD, FD or PBS treated groups. Scale bar, 85 µm. (JPG) [file pone.0066128.s006.jpg]

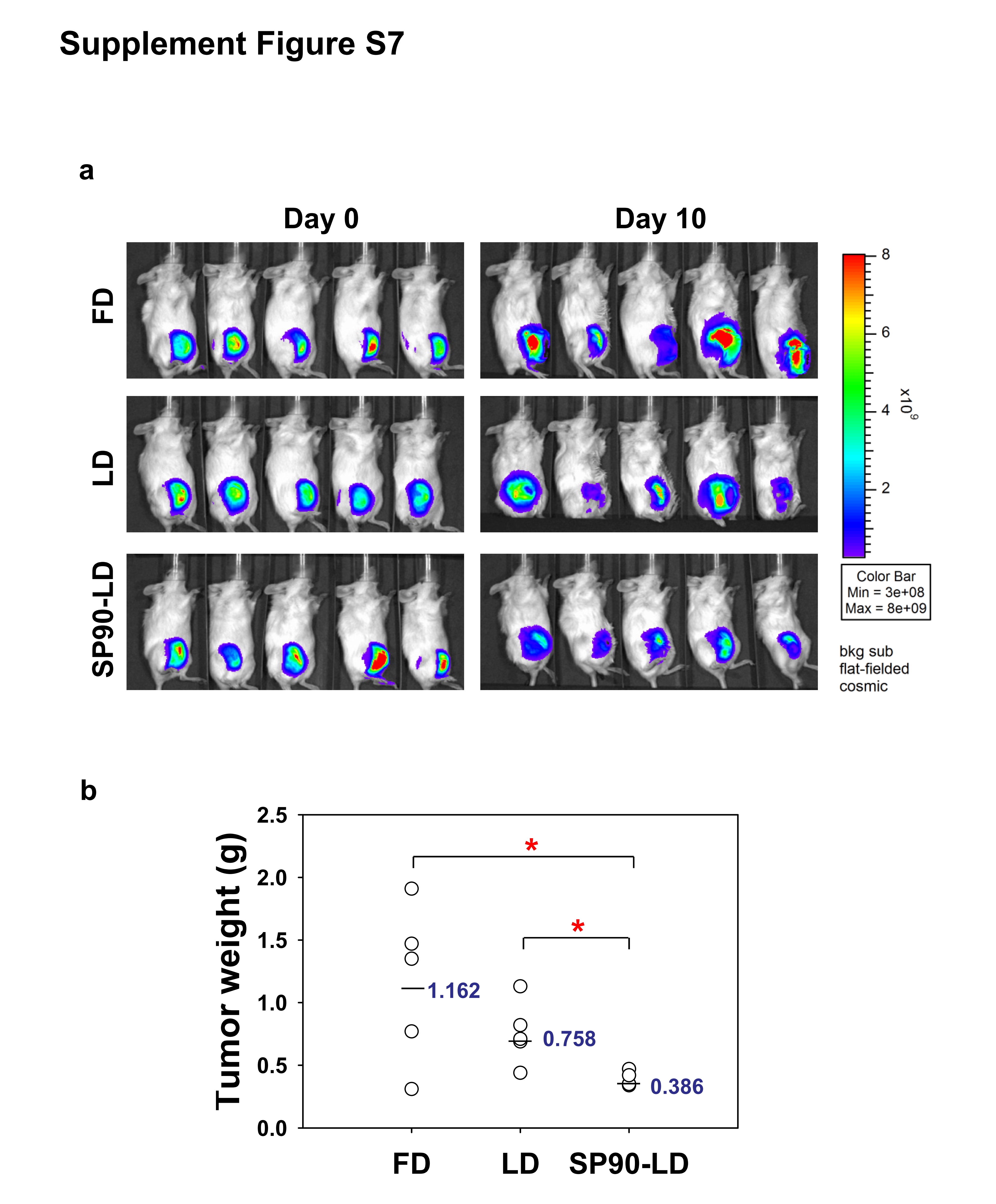

Supplement: Figure S7 — Treatment of SCID mice with SP90-LD in orthotopic human breast models. a, Representative images used for the analysis described in Figure 4c. Luminescent radiance was assessed by IVIS200 imaging on the indicated days. n = 5. b, At the end of treatment, mice were sacrificed, and tumors were dissected and weighed. *P<0.05. (JPG) [file pone.0066128.s007.jpg]

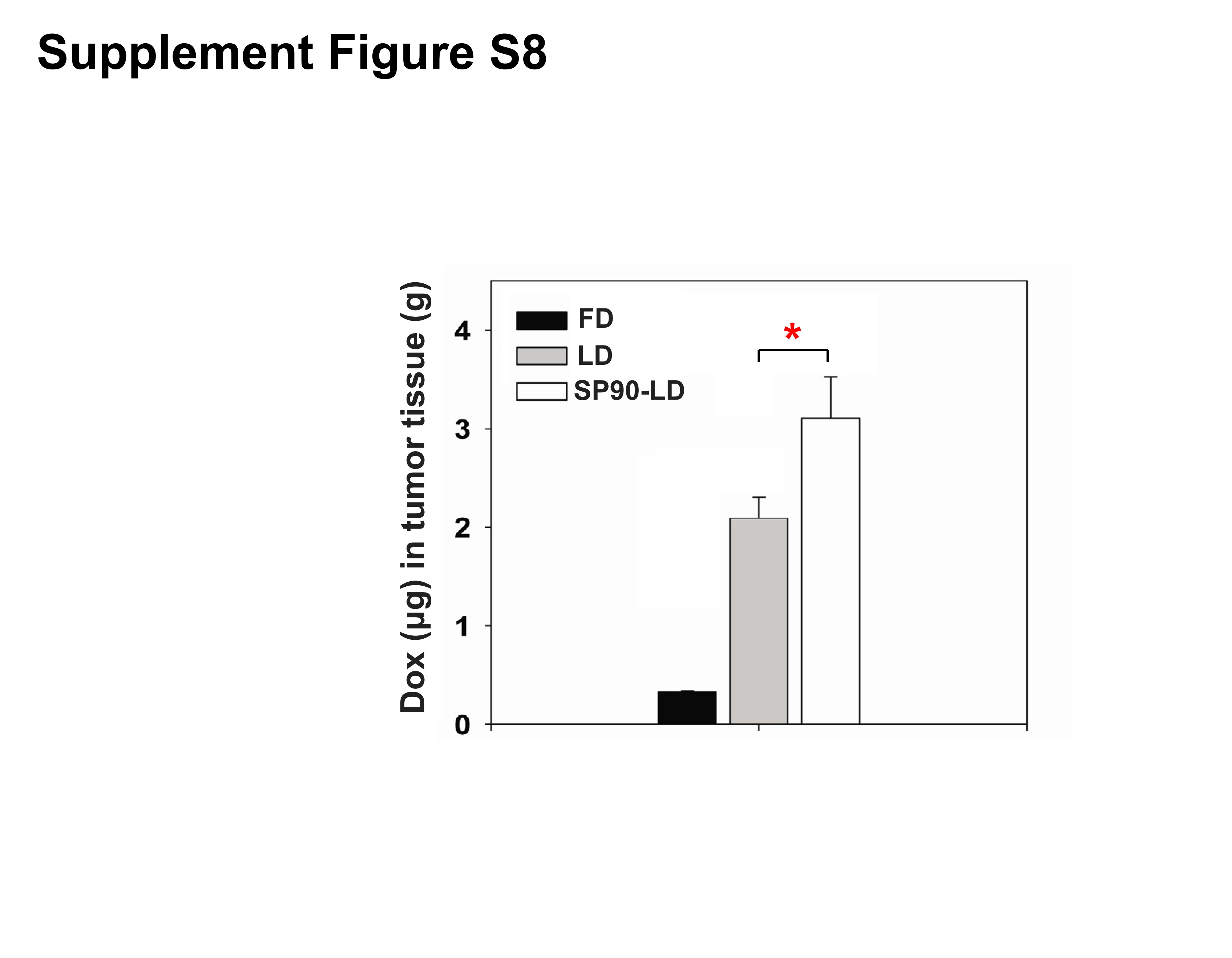

Supplement: Figure S8 — SP90-conjugated liposomes enhanced drug delivery to tumor. Accumulation of doxorubicin in tumors of breast cancer-bearing mice treated with different formulations of liposomal and free doxorubicin, without PBS perfusion (n = 3 in each group; *P<0.05). (JPG) [file pone.0066128.s008.jpg]

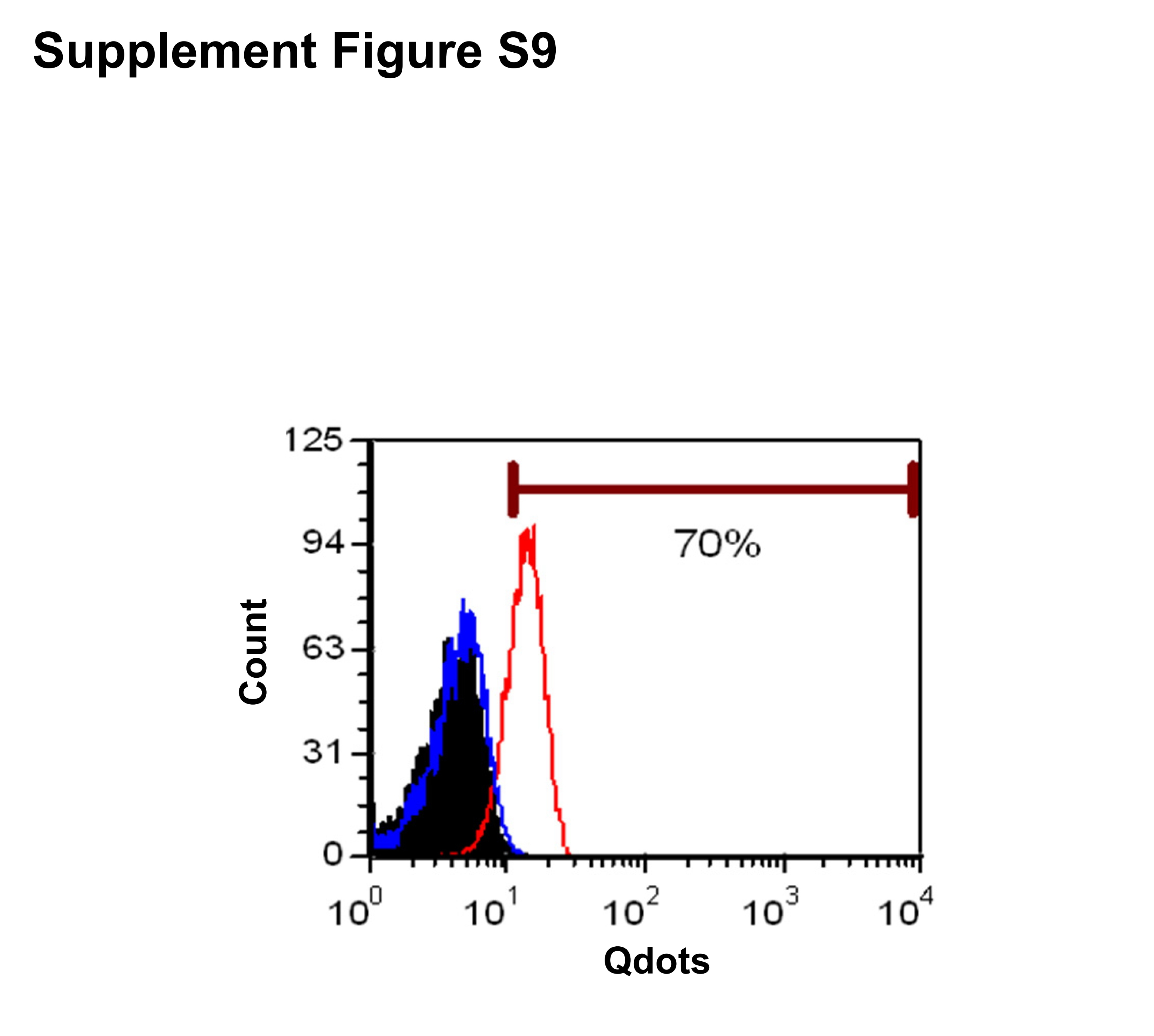

Supplement: Figure S9 — Analysis of tumor binding activity of SP90-QDs in vitro . The binding activity of QD-labeled SP90 to BT483 cells was analyzed by flow cytometry. (JPG) [file pone.0066128.s009.jpg]
