# Supplementary material for: Targeted Drug Delivery Systems Mediated by a Novel Peptide in Breast Cancer Therapy and Imaging
Source: PLoS One. 2013 Jun 11;8(6):e66128. doi: 10.1371/journal.pone.0066128 (PMC3679013; doi:10.1371/journal.pone.0066128)
Supplement: Table S1 — Detection of human breast cancer surgical specimens by PC90 phages using immunohistochemistry. (DOCX) [file pone.0066128.s010.docx]

**Supplement Tables**

**Table S1. Detection of human breast cancer surgical specimens by PC90 phages using immunohistochemistry.** (A) Prevalence of immunoreactivity of PC90 in breast cancer surgical specimens. Labeling index: 3+, more than 60% tumor cells were positive; 2+, 31-59% tumor cells were positive; 1+, less than 30% tumor cells were positive; N, no immunoreactivity was seen in the section. (B) Clinical pathological features and PC90 immunoreactivity of the human infiltrating ductal carcinoma of the breast.

**A**

| **Labeling**  **Index** | **3+** | **2+** | **1+** | **N** |
| --- | --- | --- | --- | --- |
| **Cases** | **12** | **5** | **1** | **2** |

**B**

|  | **Diagnosis** | **Tumor size** | **Tumor grading** | **Nodal involvement** | **PC90 immunoreactivity** |
| --- | --- | --- | --- | --- | --- |
| 1 | Infiltrating ductal carcinoma | pT3,  5.5 x 4.5 x 3.5 cm | Grade III, Nottingham Score: 3+2+3 points | 25/32 | 3+ |
| 2 | Infiltrating ductal carcinoma | pT2,  2.5 x 2.3 x 2.0 cm. | Grade I, Nottingham Score: 3+1+1 points | 1/9 | 3+ |
| 3 | Infiltrating ductal carcinoma | pT2,  4 x 3 x 2 cm | Grade II, Nottingham Score: 3+3+1 points | 9/21 | 3+ |
| 4 | Infiltrating ductal carcinoma | pT2,  3 x 2.5 x 2.5 cm. | Grade III, Nottingham Score: 3+3+3 points | 0/43 | 3+ |
| 5 | Infiltrating ductal carcinoma | pT1c,  2.6 x 2.1 x 1.9 cm. | Grade II, Nottingham Score: 2+2+2 points | 1/24 | 3+ |
| 6 | Infiltrating ductal carcinoma | pT4b,  5.9 x 4.9 x 3.8 cm | Grade II, Nottingham Score: 3+2+1 points | 7/21 | 3+ |
| 7 | Infiltrating ductal carcinoma | pT1c,  2 x1.7 x 1.5 cm | Grade II, Nottingham Score:: 2+3+1 points | 0/25 | 3+ |
| 8 | Infiltrating ductal carcinoma | pT2,  3 x 2.5 x 2 cm. | Grade II, Nottingham Score: 3+2+1 points | 5/29 | 3+ |
| 9 | Infiltrating ductal carcinoma | pT3,  8 x 4 x 2.5 cm | Grade II, Nottingham Score: 3+2+1 points | 23/25 | 2+ |
| 10 | Infiltrating ductal carcinoma | pT3,  5 x 3 x 3 cm | Grade II, Nottingham Score: 3+2+1 points | 8/30 | 2+ |
| 11 | Infiltrating ductal carcinoma | pT3,  5 x 4.5 x 2 cm | Grade II, Nottingham Score: 3+2+2 points | 7/22 | N |
| 12 | Infiltrating ductal carcinoma | pT2,  2 x 1.8 x 1.5 cm | Grade III, Nottingham Score: 3+3+2 points | 0/12 | 3+ |
| 13 | Infiltrating ductal carcinoma | pT2,  3.2 x 2.5 x 2 cm | Grade II, Nottingham Score: 3+2+1 points | 5/29 | 2+ |
| 14 | Infiltrating ductal carcinoma | pT2,  4.2 x 3.4 x 1 cm | Grade I, Nottingham Score: 1+2+1 points | 10/25 | 3+ |
| 15 | Infiltrating ductal carcinoma | pT2,  2.8 x 2.5 x 2.5 cm | Grade III, Nottingham Score: 3+3+3 points | 0/43 | 3+ |
| 16 | Infiltrating ductal carcinoma | pT3  7.5 x 5.3 x 2.2 cm | Grade I, Nottingham Score: 1+1+1 points | 5/15 | 2+ |
| 17 | Infiltrating ductal carcinoma | pT3,  5 x 3 x 2 cm | Grade III, Nottingham Score: 3+3+2 points | 0/1 | N |
| 18 | Infiltrating ductal carcinoma | pT2,  2.5 x 2.2 x 0.8 cm | Grade II, Nottingham Score: 3+2+2 points | 3/9 | 1+ |
| 19 | Infiltrating ductal carcinoma | pT2,  2.1x 2 x 1.9 cm | Grade II, Nottingham Score: 3+2+1 points | Cannot be assessed | 3+ |
| 20 | Infiltrating ductal carcinoma | pT2,  2.2 x 1.8 x 1.8 cm | Grade II, Nottingham Score: 2+3+1 points | 1/28 | 2+ |
