# Supplementary material for: Targeted Drug Delivery Systems Mediated by a Novel Peptide in Breast Cancer Therapy and Imaging
Source: PLoS One. 2013 Jun 11;8(6):e66128. doi: 10.1371/journal.pone.0066128 (PMC3679013; doi:10.1371/journal.pone.0066128)
Supplement: Table S2 — AUC and IC50 of BT483 treated with SP90-LD and LD. (DOCX) [file pone.0066128.s011.docx]

| **Table S2.** AUC and IC_50_ of BT483 treated with SP90-LD and LD | | |
| --- | --- | --- |
|  | **AUC** | **IC_50_** |
| **SP90-LD** | 0.92 h*μg/ml | 0.62 μg/ml |
| **LD** | 0.39 h*μg/ml | 3.06 μg/ml |
